# High-salt intake negatively regulates fat deposition in mouse

**DOI:** 10.1038/s41598-017-01560-3

**Published:** 2017-05-17

**Authors:** Huanxian Cui, Shuyan Yang, Maiqing Zheng, Ranran Liu, Guiping Zhao, Jie Wen

**Affiliations:** 1 Institute of Animal Sciences, Chinese Academy of Agricultural Sciences, Beijing, 100193 China; 2 State Key Laboratory of Animal Nutrition, Beijing, 100193 China; 3 Institute of Zoology, Chinese Academy of Sciences, Beijing, 100101 China

## Abstract

High-salt (HS) intake contributes to hypertension and cardiopathy, but the effect of HS on fat deposition is controversial. Feed intake, fat mass, the percentage of abdominal fat, heat production, rate of oxygen consumption and the respiratory exchange ratio of mice on a HS diet were significantly decreased (P < 0.01 or 0.05) compared with mice on a normal-salt (NS) diet. An *in vitro* experiment with differentiating pre-adipocytes showed reduced fat deposition in the presence of high concentrations of NaCl (>0.05 M). Abdominal fat mRNA profiles and protein measurements showed that 5 known genes involved in lipolysis were up-regulated significantly and 9 genes related to lipogenesis were down-regulated in HS mice. Abundant genes and some proteins (ATP2a1, AGT, and ANGPTL4) related to calcium ion metabolism or the renin-angiotensin system (RAS) were differentially expressed between HS and NS mice. Of special interest, CREB1 phosphorylation (S133 and S142), a key factor involved in calcium signaling and other pathways, was up-regulated in HS mice. By IPA analysis, a network mediated by calcium was established providing the molecular mechanisms underlying the negative effect of HS on fat deposition.

## Introduction

The growth of white adipose tissue (WAT) involves an increase in number of fat cells and an increase in the storage of triacylglycerol (TAG) in lipid droplets inside adipocytes. Lipid mobilization from adipocytes, lipolysis, consists of TAG hydrolysis and the release of free fatty acids and glycerol. These processes are regulated by hormones and represent an important mechanism for controlling WAT mass^1^. Adipocytes act as endocrine cells and secrete a wide variety of hormones, such as leptin, an adipocyte-derived hormone that regulates appetite and energy expenditure. Leptin gene expression and plasma concentration reflect body fat content^2, 3^.

Recently, high-salt (HS) intake has received increased attention because of its negative effects on human health. For example, HS intake contributes to hypertension and heart disease^4–9^. The role of HS on lipid metabolism and fat deposition is currently debated. A low salt diet can induce fat deposition in the wall of large arteries and cause atherosclerosis^10^ but HS intake increases plasma leptin concentrations and induces hypertrophy of fat cells, which may in increased white fat volume^11^, whereas low salt helps reduce obesity^12^. A separate study shows conflicting results where a HS diet reduced food intake by 10 to 15% and body fat was considerably reduced^13^. To clarify the effect of HS intake on fat deposition and to delineate the underlying regulatory mechanism, mice were fed a HS diet and the 3T3-L1 cell line was used. The results highlight a novel pathway involved in HS intake and fat deposition.

## Results

### HS intake reduces fat deposition in mouse adipose tissue

**Figure 1:**
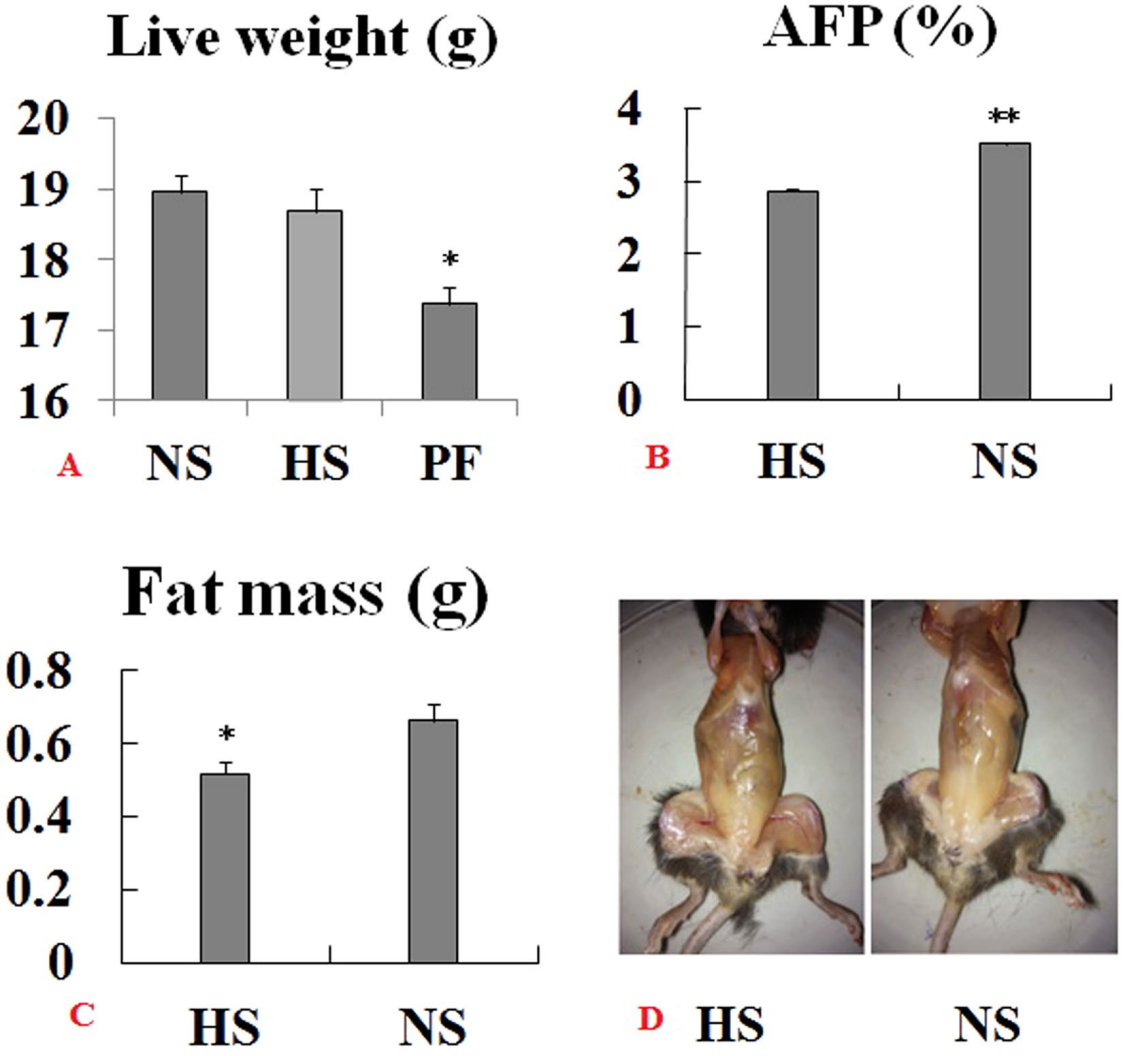
Change in fat deposition in adipose tissue. Mice were fed a HS (4% NaCl) or a NS (0.4% NaCl) diet or pair-fed (PF) with NS at the level of HS consumed for 8 wk. (**A**) live weight; (**B**) AFP (% of liveweight); (**C**) abdominal fat mass measured; and (**D**) pictured. Data are means ± SEM, n = 10. *P < 0.05; **P < 0.01.

**Figure 2:**
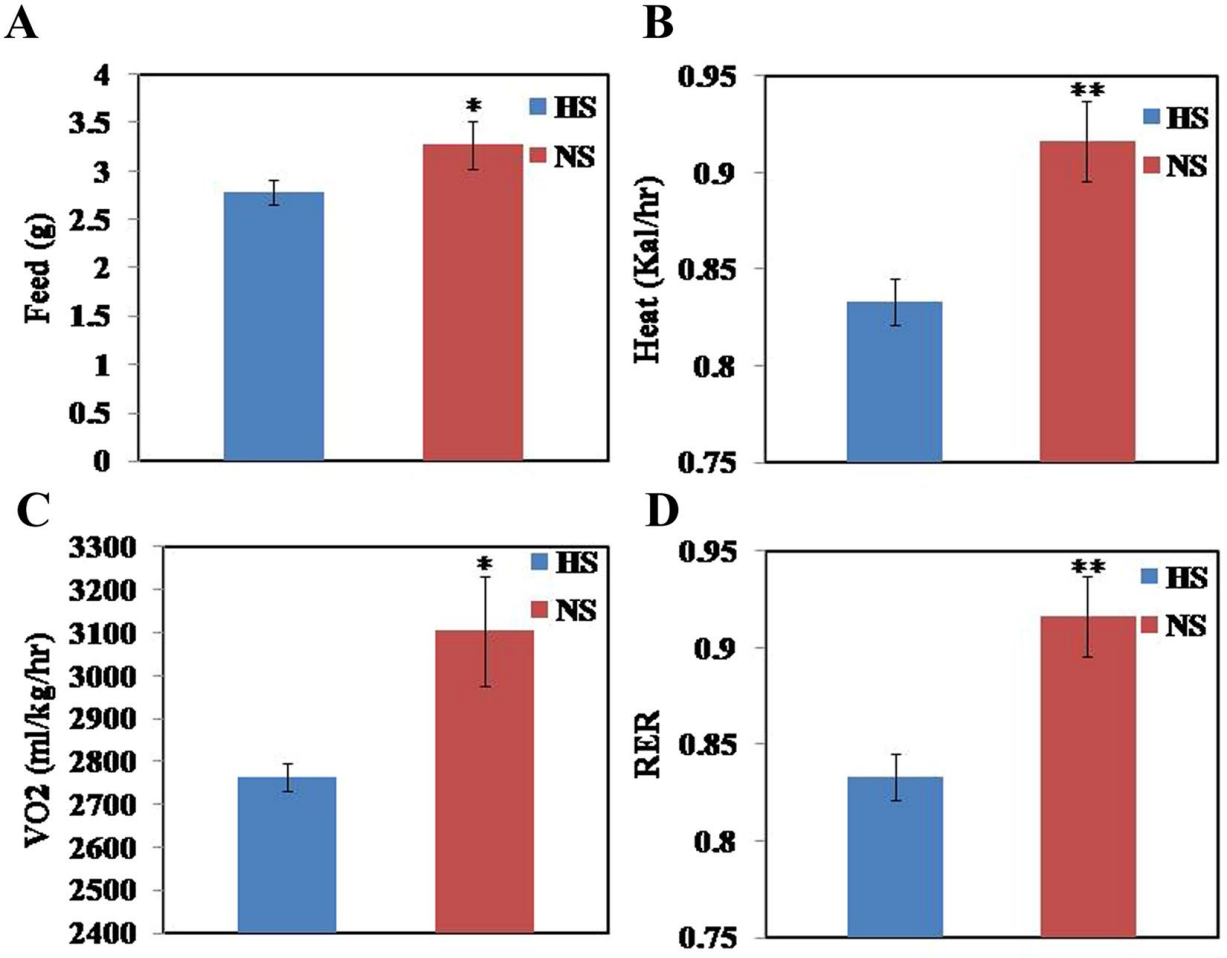
Changes in feed intake and body metabolism. Feed intake and body metabolism were measured after mice were fed a HS (4% NaCl) or NS (0.4% NaCl) diet for 8 wk. (**A**) Feed intake (g), (**B**) heat production (kcal/hr), (**C**) VO_2_ (oxygen consumption, ml/kg/hr), and (**D**) respiratory exchange rate (RER). Data are means ± SEM, n = 8. *P < 0.05; **P < 0.01.

To determine if HS intake plays a role in fat deposition, a HS diet (4% NaCl) and a NS (0.4% NaCl) were fed ad libitum to female mice for 8 wk. Live weight and fat mass were recorded, and the percentage of abdominal fat (AFP) was calculated. Live weight (feed withdrawn for 18 h) of HS mice decreased by 4.14% compared with NS mice (Fig. 1A), but the difference was not significant (P > 0.05). The fat mass of HS mice was significantly decreased (21.96%) compared to NS mice (Fig. 1C,D, P < 0.01); similarly, AFP (HS 2.86%, NS 3.51%) was significantly decreased by 18.52% (P < 0.01, Fig. 1B). The results showed that HS intake negatively regulated abdominal fat deposition of female mice. Feed intake and body metabolism were measured for 2 d using CLAMS after HS and NS diets were fed for 8 wk. Feed intake of HS was significantly lower than NS (P < 0.05, Fig. 2A). Similarly, heat production, VO_2_, and RER in the HS mice were all significantly decreased (P < 0.01, P < 0.05, P < 0.01, respectively, Fig. 2B–D) indicating that HS intake down-regulated feed intake and reduced whole body metabolism in mice.

Because of the reduced intake of the HS diet, a pair-fed (PF) control group was used to account for possible interference from taste aversion because of the high salt. Compared to the NS mice and HS mice, live weight in the PF mice was also significantly lower (P < 0.05, P < 0.05, Fig. 1A), showing that possible taste aversion little negative impact on whole body growth.

**Figure 3:**
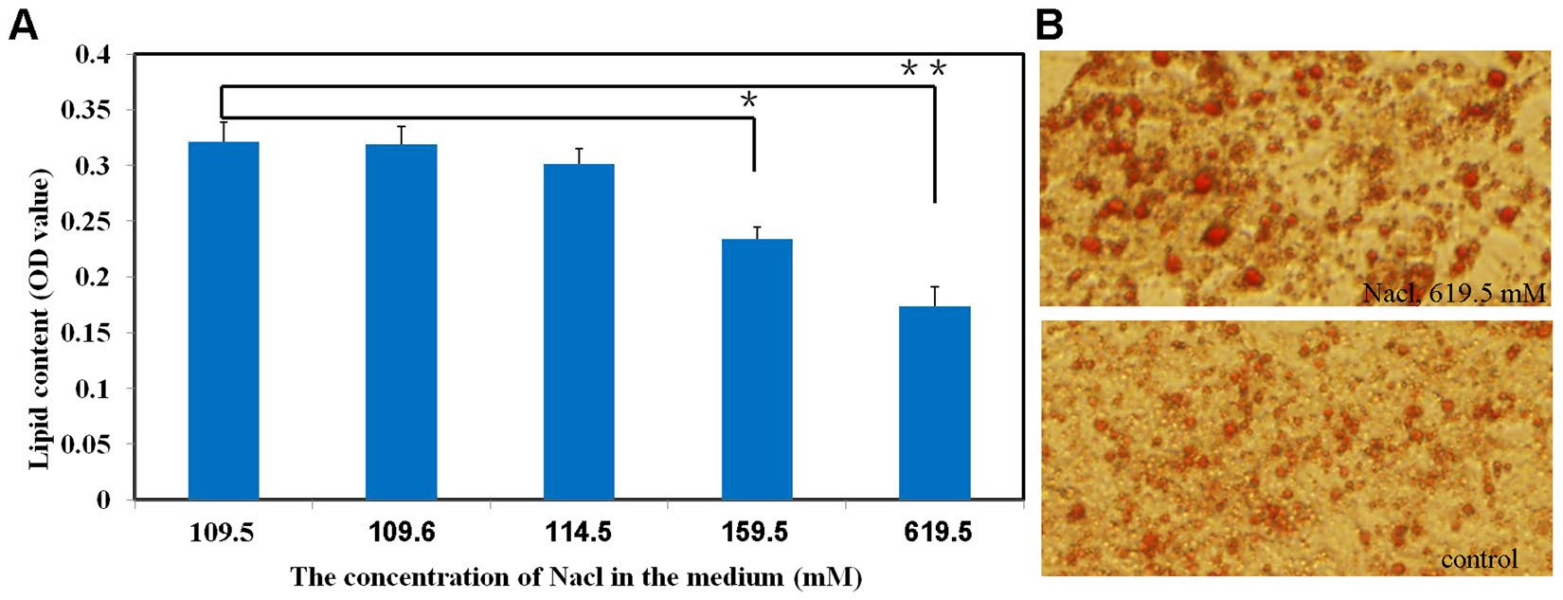
The effect of NaCl on lipid content in 3T3-L1 cells. Cells were examined, 10 d after inducing differentiation, in media containing 0, 0.0005 M, 0.005 M, 0.05 M, or 0.5 M additional NaCl. (**A**) The lipid content significantly decreased with 0.05 M and 0.5 M additional NaCl compared with controls. Data are means ± SEM, n = 6. *P < 0.05; **P < 0.01. (**B**) Morphological changes and lipid deposition induced by 0.5 M additional NaCl (200×).

To explore the link between HS intake and fat deposition, direct effects of increased NaCl concentrations on the lipid content of 3T3-L1 cells, induced to differentiate and examined after 10d. With increasing total NaCl concentration, the lipid contents gradually decreased, and had the significant differences (P < 0.05, P < 0.01, respectively) in cells with 159.5 and 619.5 mM NaCl treatments (50 and 500 mM supplemented) compared to control cells with 109.5 mM NaCl treatment (Fig. 3A,B). The results illustrated that high concentrations of additional NaCl (>50 mM) inhibited intracellular fat accumulation in differentiated 3T3-L1 cells.

### HS intake negatively regulates fat deposition in mouse

**Figure 4:**
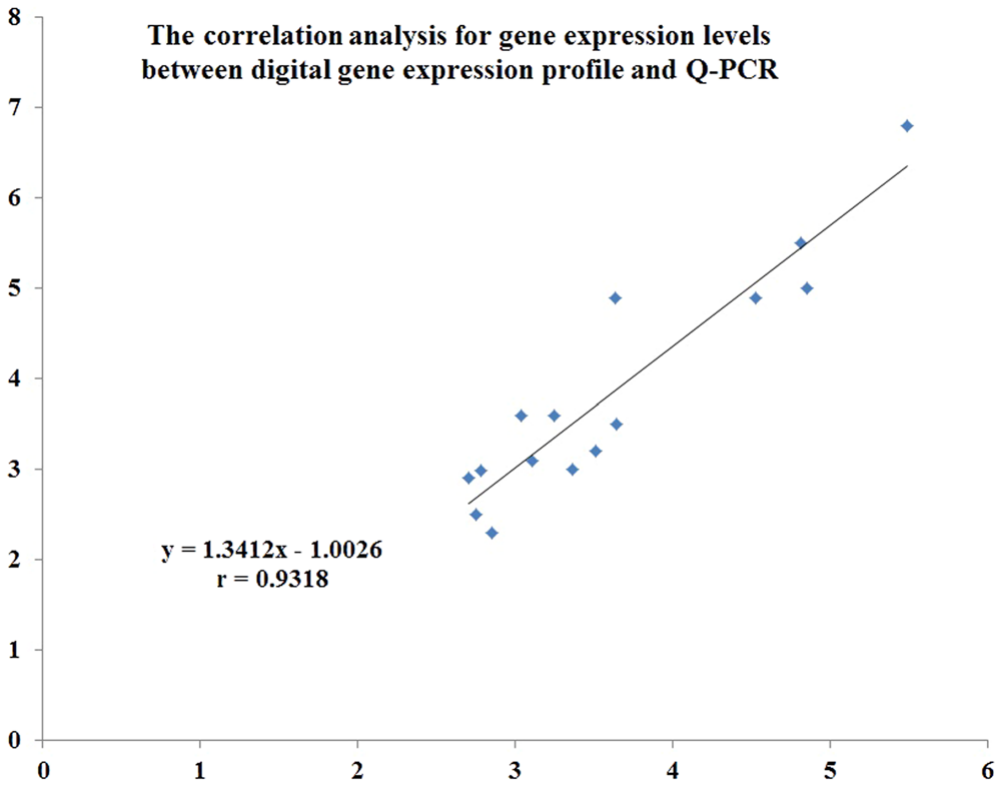
Technical validation of digital gene expression profile results by q-PCR. The r value (r = 0.932) indicates Spearman’s Correlation between the 2 methods.

**Figure 5:**
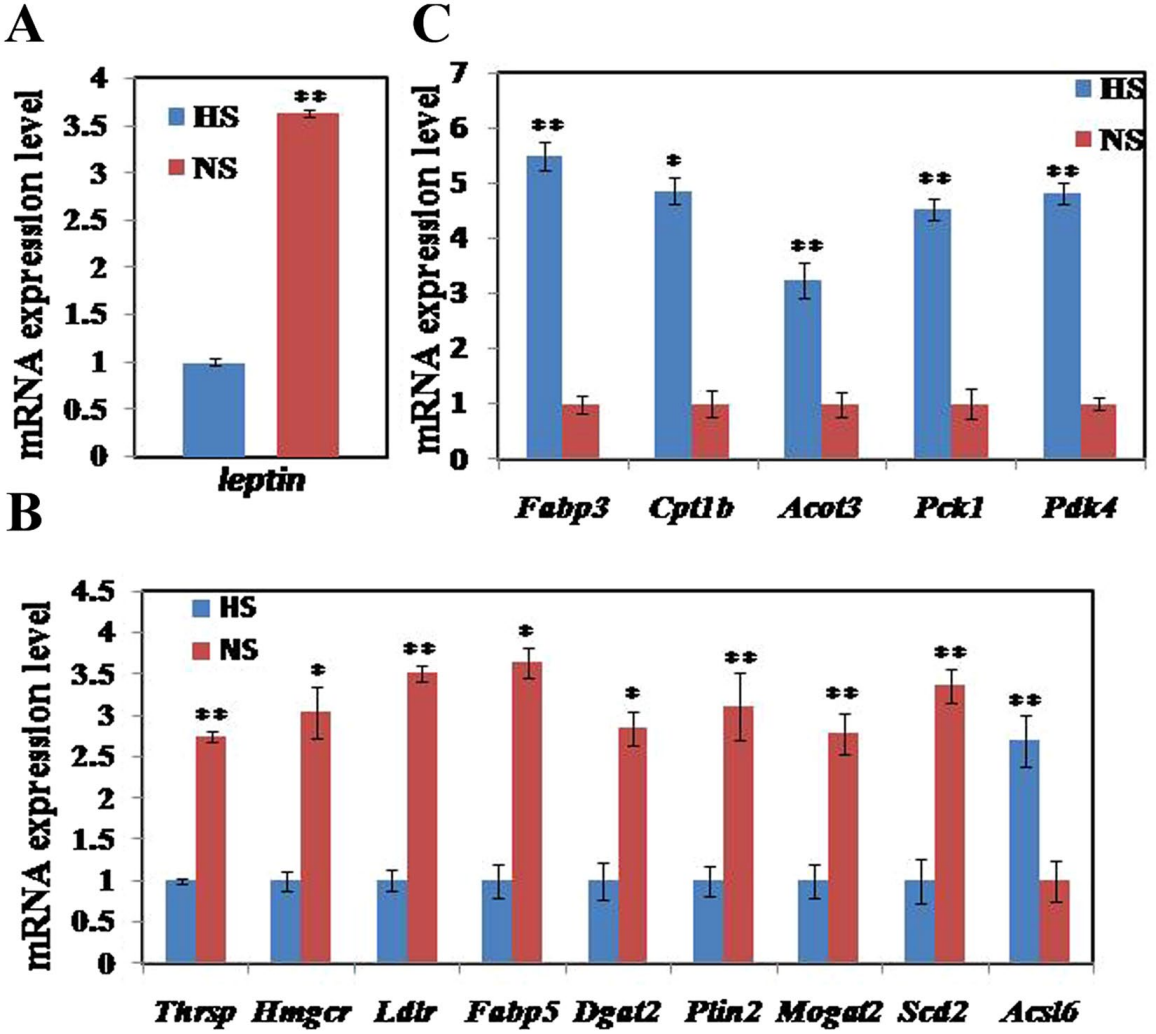
Changes in mRNA abundance of genes related to feed intake and lipid metabolism in adipose tissue after mice were fed 8 wk with HS and NS. (**A**) mRNA levels of lep, which is involved in food intake, was down-regulated. (**B**) mRNA levels of genes, involved in lipogenesis were down-regulated. (**C**) mRNA levels of genes involved in lipolysis or carbohydrate utilization were up-regulated. Data are means ± SEM, n = 3. *P < 0.05; **P < 0.01.

A total of 565 known differentially expressed genes (DEGs, fold change ≥ 1.5) were identified in abdominal fat from mice fed HS and NS diets; 362 were up-regulated and 203 were down-regulated (Additional file 2). Based on those known DEGs, the enriched GO-terms (Additional file 3) and KEGG pathways (Additional file 4) were also identified. Numerous DEGs were enriched in the calcium ion, glycolysis/gluconeogenesis, PPAR and HIF-1 signaling pathways, which might contribute to the effect of the HS diet on fat deposition. Ninety-one DEGs involved in lipid metabolism were identified (Additional file 5). To confirm results from the digital gene expression profile, 15 representative genes related to lipid metabolism were compared with q-PCR in adipose tissue from the 2 diets. As shown in Fig. 4, fold-changes in gene expression using the 2 methods were highly correlated (r = 0.932), demonstrating that the results of digital gene expression profiling have sufficient accuracy. The abundance of leptin transcripts in mice fed HS was significantly decreased (P < 0.01) compared with the NS mice, as confirmed by q-PCR (Fig. 5A). It indicated that mice in the HS group had reduced feeding or were in a low digestive state. In addition, transcript abundance by q-PCR of some genes related to lipogenesis (Thrsp, Scd2, Hmgcr, Ldlr, Dgat2, Mogat2, Plin2, and Fabp5) in the HS mice were significantly decreased (P < 0.05 or P < 0.01, Fig. 5B), and the expression of Acsl6 in the HS mice was significantly increased (P < 0.01, Fig. 5B). On the other hand, the mRNA levels of some genes involved in lipolysis or carbohydrate utilization (Fabp3, Cpt1b, Acot3, Pck1, and Pdk4) were significantly increased in HS mice (P < 0.05 or P < 0.01, Fig. 5C). These results indicated that HS decreased lipogenesis and increased lipolysis or carbohydrate utilization when compared with the NS. This is in agreement with the observed traits where the HS diet reduced feed intake, body metabolism and fat deposition.

### Calcium signaling mediates the effect of HS on food intake and lipid metabolism

**Figure 6:**
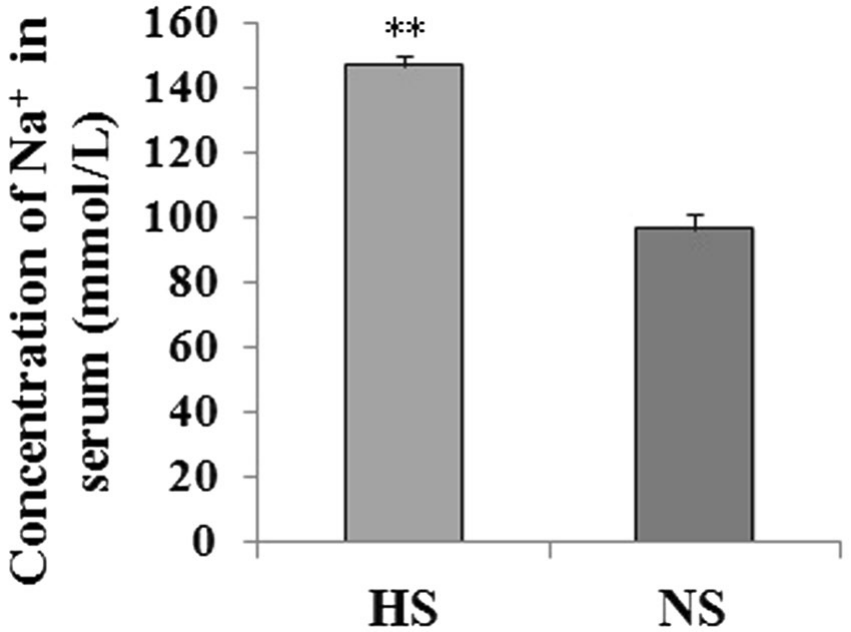
Sodium ion concentration in serum from mice fed HS and NS diets. The sodium ion concentration in serum was significantly higher (P < 0.01) in HS mice. Data are means ± SEM, n = 6, **P < 0.01.

**Figure 7:**
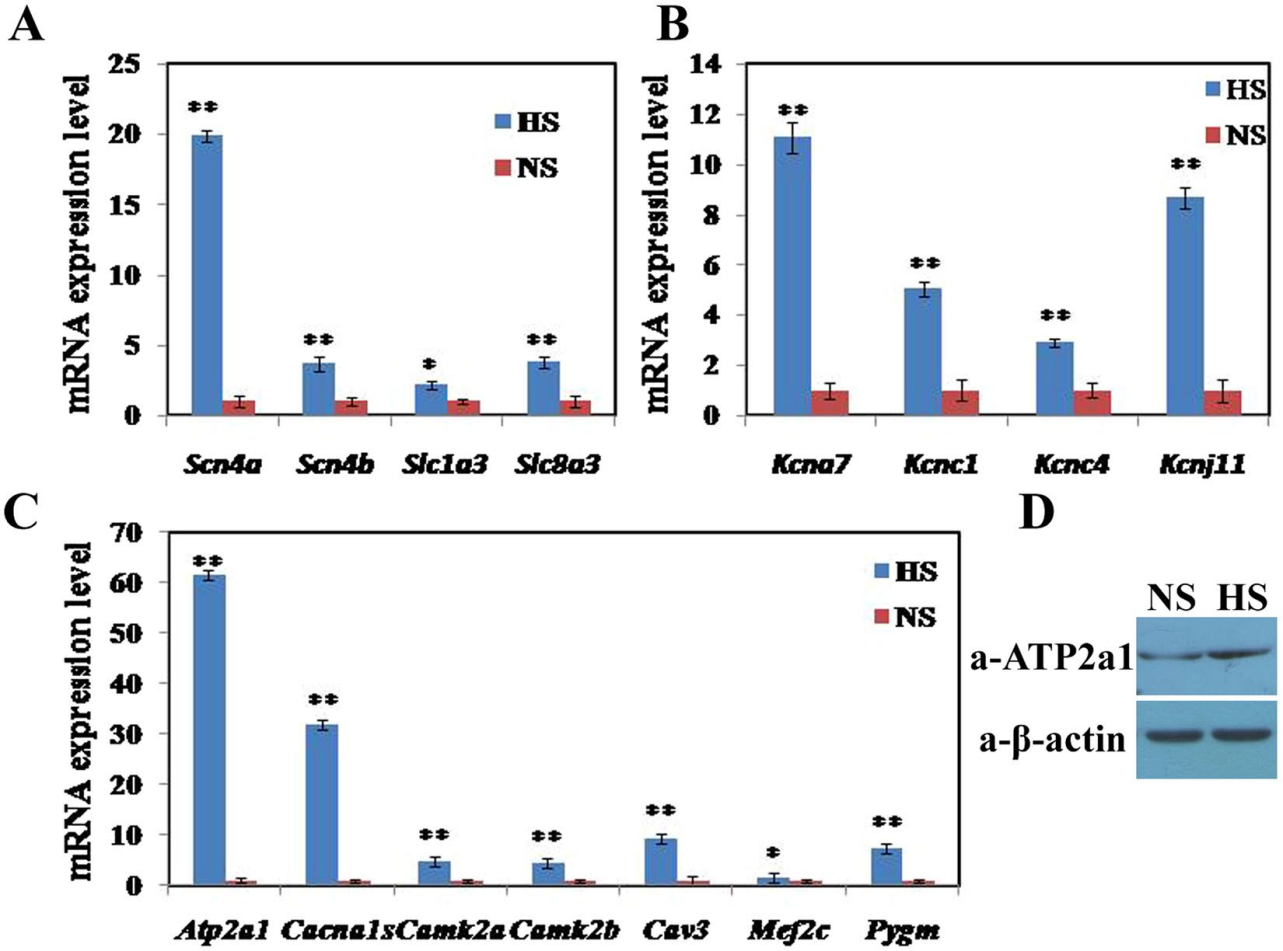
Up-regulated expression of genes related to sodium, potassium and calcium ion metabolism, and protein level of ATP2a1 in adipose tissue after mice were fed for 8 wk with HS. (**A**) mRNA levels of genes related to sodium ion metabolism were up-regulated. (**B**) mRNA levels of genes related to potassium ion metabolism were up-regulated. (**C**) mRNA levels of some genes related to calcium ion and lipid metabolism were up-regulated. (**D**) The protein content of ATP2a1 was increased. Data are means ± SEM, n = 3. *P < 0.05; **P < 0.01.

The sodium ion concentration in serum of HS mice was significantly higher (P < 0.01) than that in NS mice (Fig. 6) and the gene expression profiling differential expression of genes related to sodium in adipose tissue (Additional file 6) after 8 wk of feeding the HS diet; some representative genes were: Scn4a, Scn4b, Slc1a3 and Slc8a3, and their relative expression in HS mice was significantly up-regulated (P < 0.05 or P < 0.01) compared with the NS mice (Fig. 7A).

According to the DEGs between mice fed HS and NS diets, calcium signaling pathway was enriched (Additional file 4). The relative expression of genes involved in potassium ion metabolism (Kcna7, Kcnc1, Kcnc4 and Kcnj11) were significantly up-regulated (P < 0.01) in HS mice (Fig. 7B). Similarly, genes related to calcium ion metabolism (Agt, Atp2a1, Cacna1s, Cacng1, Camk2a, Camk2b, Capn11, Capn3, Casq1, Cav3 and Csrp3, *et al*.) were significantly increased in HS mice (P < 0.05 or P < 0.01, Additional file 6). Seven genes (Atp2a1, Cacna1s, Camk2a, Camk2b, Cav3, Mef2c and Pygm), as the intersection between lipid metabolism and calcium metabolism, were identified and had 1.60- to 61.61-fold change (Fig. 7C). Further, the quantity of the ATP2a1 protein in adipose tissue of HS mice was also significantly increased (P < 0.05) compared with NS mice (Fig. 7D). These results indicated that calcium signaling had mediated the regulating of lipid metabolism by HS intake.

**Figure 8:**
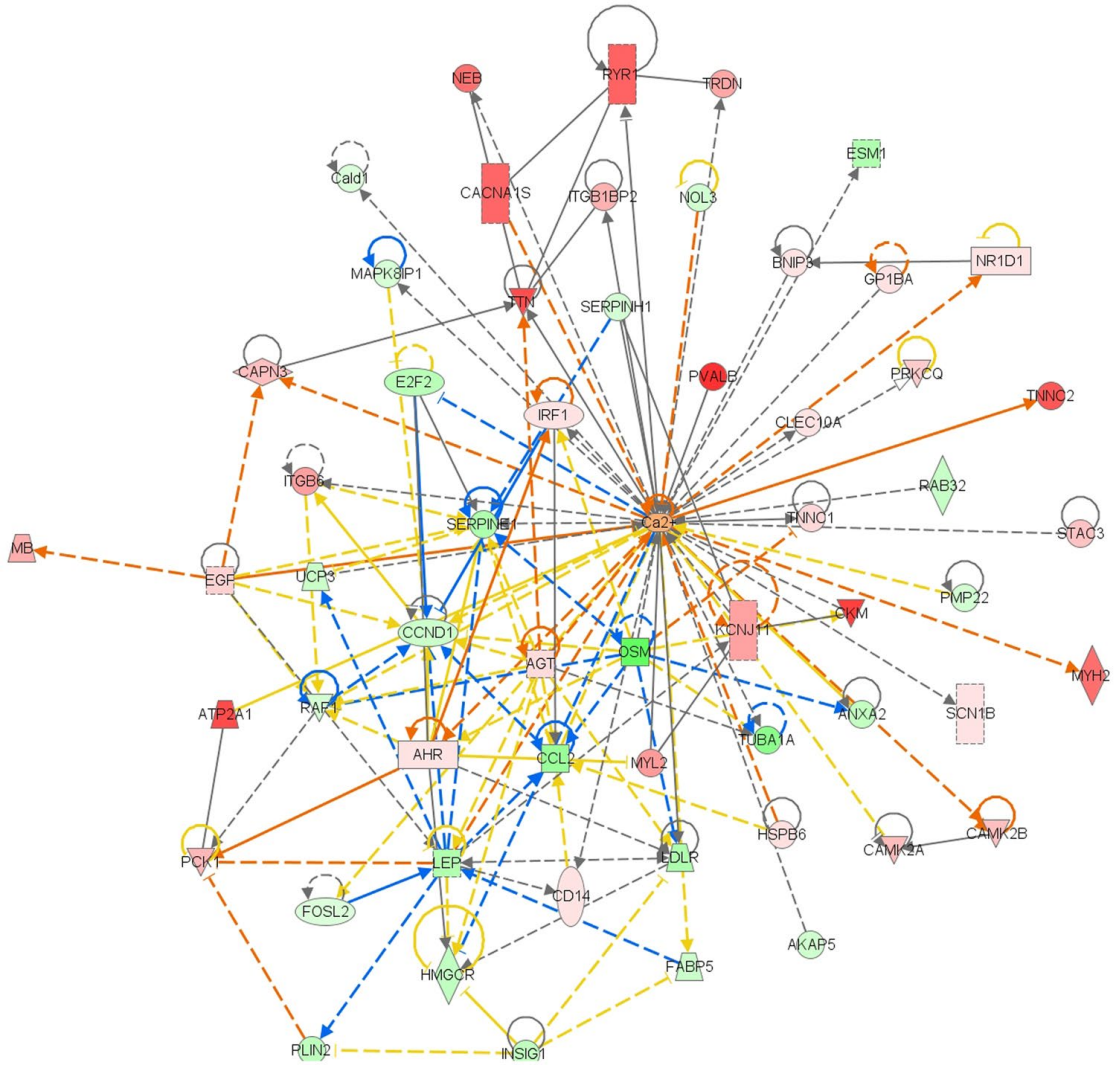
The mechanistic regulatory network, based on calcium ion and DEGs identified between HS group and NS group, by IPA software.

To clarify the relationship of calcium ion and lipid metabolism, calcium ion combined with DEGs was imported into the regulatory network using IPA software (Fig. 8). The results showed that calcium both directly regulated factors involved in the calcium ion pathway (including Atp2a1, Cacna1s, Camk2a, Camk2b, and Capn3), and regulated genes of lipid metabolism (Lep, Hmgcr, Ldlr and Fabp5). This indicated that calcium participated in the expression of genes related to lipid metabolism and inhibition of fat deposition. In addition, there was interaction among Lep, Hmgcr, Ldlr and Fabp5 genes in lipid metabolism; calcium ion inhibited intake by the change expression of Lep and there was a negative feedback mechanism involving Lep on the expression of Ldlr and Fabp5. The regulatory mechanism for HS influencing lipid metabolism was shown to be complicated but, clearly, the HS diet affected feed intake and lipid metabolism to reduce fat deposition in adipose tissue.

### Renin-angiotensin system (RAS) regulates HS influence on fat deposition in adipose tissue

**Figure 9:**
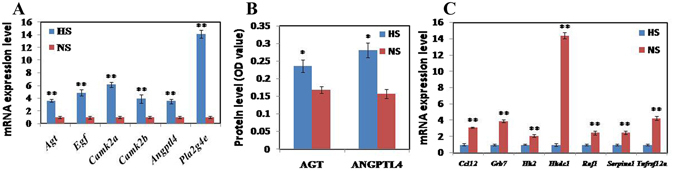
Changes in the mRNA and protein levels of genes involved in RAS signaling in adipose tissue from feeding a HS diet. (**A**) Transcript abundance of genes, which are jointly involved in RAS and lipid metabolism, were up-regulated. (**B**) The protein contents of AGT and ANGPTL4 in abdominal fat (AF) were increased. (**C**) Transcript abundance of genes, which are jointly involved in RAS and lipid metabolism, were down-regulated. Data are means ± SEM, n = 3. *P < 0.05; **P < 0.01.

Twenty DEGs involved in RAS (Additional file 7) were identified. Among them, Agt, Egf, Camk2a, Camk2b, Angptl4, Pla2g4e, Ccl12, Grb7, Hk2, Hkdc1, Raf1, Serpine1, and Tnfrsf12a were found at the intersections between RAS and lipid metabolism. The expression of Agt, Egf, Camk2a, Camk2b Angptl4 and Pla2g4e by q-PCR was significantly up-regulated in tissue from HS mice (P < 0.01) compared with the NS mice (Fig. 9A). Tissue contents of AGT and ANGPTL4 protein were significantly increased in adipose tissue from mice on HS diet (P < 0.05, P < 0.05, respectively, Fig. 9B). The relative abundance of Ccl12, Grb7, Hk2, Hkdc1, Raf1, Serpine1, and Tnfrsf12a transcripts in adipose tissue of the HS mice were significantly down-regulated (P < 0.01, Fig. 9C); these changes were consistent with reduced fat deposition from high-salt intake. From Additional file 7, Agt, Egf, Camk2a, Camk2b, Acta1, Actn2, Actn3 and Prkcq had intersection with calcium ion metabolism. Given the role of calcium ion in lipid metabolism noted above, the DEGs involved in RAS might play a role in reducing fat deposition of HS mice via calcium ions.

### CREB1 regulates fat deposition in adipose tissue of mice on HS diet

**Figure 10:**
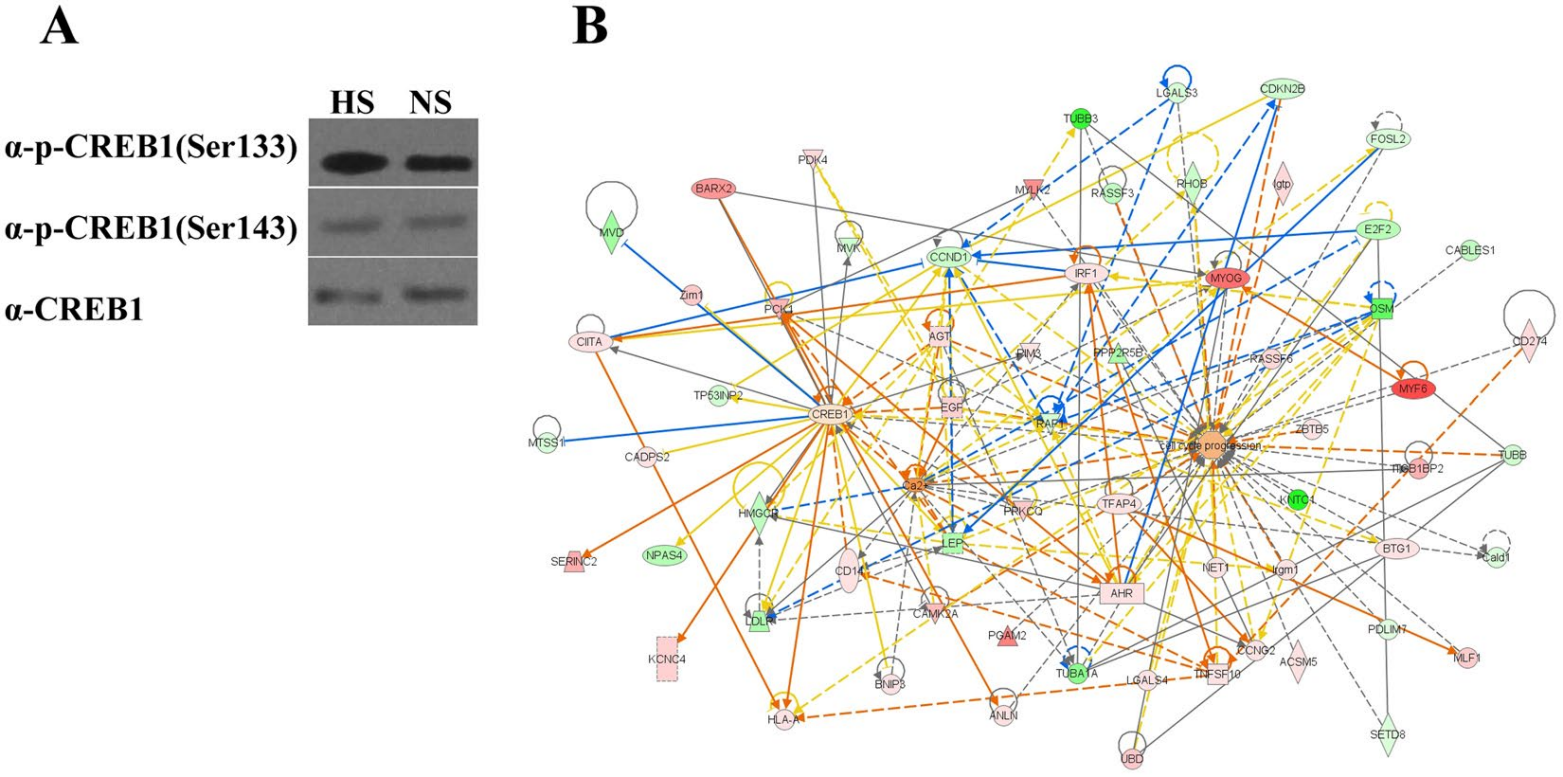
Phosphorylation of CREB1 protein (**A**) and the regulatory network based on calcium ion, CREB1 and DEGs by IPA software (**B**).

CREB1 has two phosphorylation sites (S129, S133 and S142) and, because their phosphorylation is known to play a regulatory role in these pathways, the phosphorylation of CREB1 was analyzed here. Both ser133 and ser142 were phosphorylated to a greater extent in the HS mice compared with the NS mice (Fig. 10A), indicating enhanced activity of CREB1 caused by HS intake.

According to the DEGs between mice fed HS and NS diets, 11 pathways were enriched (Additional file 4), including cardiopathy, calcium signaling, PPAR signaling, glycolysis/gluconeogenesis, tight junction and HIF-1 signaling. Based on Fig. 8, CREB1 was then imported into the regulatory network using IPA software to illuminate the relationships with calcium ion and the DEGs. From Fig. 10B, CREB1 would directly regulate expression of genes related to lipid metabolism, including Lep, Ldlr and Hmgcr yet CREB1 is also regulated by Agt and Egf, involved in the RAS. Meanwhile, CREB1 is also directly regulated by calcium ion and related genes such as Camk2a and Prkcq. All results indicated that CREB1 had an important regulating role in the lipid metabolism process, and genes in the RAS and calcium ion or its metabolism regulate lipid metabolism in adipose tissue through CREB1 because of HS intake.

## Discussion

It is known that HS intake is harmful to human health and contributes to hypertension, heart disease, and arteriosclerosis, yet the effect of HS intake on fat deposition is not clear^10–13^. A major goal of this study was to better understand the effect of HS intake on fat deposition in adipose tissue and to delineate a regulatory mechanism. Accordingly, mice were fed a HS diet to examine this at the whole body level and the 3T3-L1 cell line was used to better expose mechanisms at the cellular and molecular level.

### HS intake inhibits fat deposition in mouse adipose tissue

Following ad lib feeding of the HS diet for about 8 wk, the reduction in live weight was not significant whereas fat mass and AFP was significantly decreased. These results support the idea that HS negatively regulates fat deposition in adipose tissue of female mice and extends an earlier finding in male rats that HS intake reduces fat mass^13^. In the present study, feed intake, heat production, VO_2_ and RER were reduced in mice fed the HS diet.

Growth of WAT results from increased numbers and hypertrophy of adipocytes. To further explore the effect of HS on fat deposition, 3T3-L1 cells were cultured in increasing concentrations of NaCl. Cell differentiation, as measured by Oil Red O, showed that the storage of lipid within adipocytes was reduced with high concentrations of NaCl but there was no significant difference in cell numbers (P > 0.05, data not shown). This result indicated that HS intake inhibited intracellular fat deposition by impairing the process of adipocyte differentiation.

On a molecular level, calcium signaling, glycolysis/gluconeogenesis, PPAR signaling and HIF-1 signaling were all enriched with HS. Previous studies^14–17^ indicate that these pathways could directly or indirectly impact lipid metabolism. In adipose tissue, 91 known DEGs involved in lipid metabolism were identified. Among them, Lep, a key factor in appetite, can inhibit feed intake^18, 19^ and Lep mRNA was significantly decreased by the HS diet. Genes related to lipogenesis were significantly decreased in mice fed the HS diet, including those involved in fatty acid and lipid biosynthesis and transport (Scd2, Fabp5, Thrsp and Hmgcr)^20–23^, lipid endocytosis (Ldlr)^24^, and lipid esterification (Dgat2, Mogat2 and Plin2)^25–27^. Acsl6 is a negative regulator of lipid metabolism^28^. Conversely, genes related to lipolysis and fatty acid decomposition (specifically, Fabp3, Cpt1b, and Acot3)^29–31^, or carbohydrate utilization (Pck1 and Pdk4)^32, 33^ were significantly up-regulated by the HS diet. All of these genes are involved in these 11 screened KEGG pathways in this study. These results were consistent with the HS diet depressing feed intake and lipogenesis and enhancing lipolysis, thereby contributing to reduced fat deposition in mouse adipose tissue.

Results obtained from whole body, cellular, and molecular experiments confirms the idea that HS reduces feed intake and, more strikingly, reduces fat deposition.

### HS regulates fat deposition through calcium signaling, RAS, and CREB1

Gene expression profiling was examined further to illuminate the molecular mechanism underlying the effect of HS on fat deposition. Genes related to sodium metabolism (Scn4a, Scn4b, Slc1a3, and Slc8a3) and potassium metabolism (Kcna7, Kcnc1, Kcnc4, and Kcnj11) were significantly up-regulated by HS intake for 8 wk. Similarly, genes related to calcium ion metabolism (Agt, Atp2a1, Cacna1s, Cacng1, Camk2a, Camk2b, Capn11, Capn3, Casq1, Cav3 and Csrp3, *et al*.) were significantly increased in HS mice. Seven DEGs (Atp2a1, Cacna1s, Camk2a, Camk2b, Cav3, Mef2c and Pygm) involved in calcium and lipid metabolism were also identified and their relative expression was significantly up-regulated in mice fed the HS diet (1.60- to 61.61-fold change). In addition, as the key factor in calcium ion transport^34^, both mRNA abundance and the amount of ATP2a1 protein in adipose tissue were significantly increased by HS intake. These results indicate that HS intake induces the exuberant mediation of calcium signaling pathway in adipose tissue, and the enhancement of calcium ion activity in adipose tissue of HS mice was probably induced by the Na^+^-K^+^ pump.

According to the regulatory network generated by IPA software, as shown in Fig. 8, calcium directly regulates genes involved in the calcium pathway (including Atp2a1, Cacna1s, Camk2a, Camk2b, and Capn3), those involved in appetite and lipid metabolism (Lep, Hmgcr, Ldlr, and Fabp5). The results strongly indicate that calcium ion participates in regulating lipid metabolism, and negatively regulates food intake and fat deposition in adipose tissue in response to HS intake. In addition, interactions between Lep, Hmgcr, Ldlr, and Fabp5, indicate a negative feedback mechanism from Ldlr and Fabp5 with expression of Lep, showing the complicated regulatory mechanism of lipid metabolism involving both intake and cellular lipid metabolism in reducing fat deposition in adipose tissue in response to HS intake.

It is interesting that Agt, Egf, Camk2a, Camk2b, Angptl4, Pla2g4e, Ccl12, Grb7, Hk2, Hkdc1, Raf1, Serpine1, and Tnfrsf12a were found at the intersection between the RAS and lipid metabolism. The expression of Agt, Egf, Camk2a, Camk2b, Angptl4 and Pla2g4e was significantly up-regulated by HS intake, and that of Ccl12, Grb7, Hk2, Hkdc1, Raf1, Serpine1, and Tnfrsf12a significantly down-regulated. Both Changes in mRNA and protein levels of Agt and Angptl4 were consistent. Among these, Agt and Egf may promote differentiation of preadipocytes^35, 36^ and Angptl4 may inhibit fat deposition by depressing LPL activity^37^. Figure 8 showed that Agt directly inhibits the expression of Lep, Hmgcr, and Ldlr and Egf negatively regulates the expression of Lep. Conversely, Lep and Hmgcr could regulate the expression of Ccl12. The results show that HS intake reduces fat deposition in mouse adipose tissue through RAS genes. Figure 8 also showed that Agt and Egf positively regulate calcium ion metabolism and genes involved in RAS signaling also overlap with calcium ion metabolism. Specifically, expression of Agt, Egf, Camk2a, and Camk2b was significantly up-regulated by HS intake. And these genes are also involved in lipid metabolism. As previously discussed, it is hypothesized that calcium plays a regulatory role in lipid metabolism and now it can be further hypothesized that genes involved in RAS regulate lipid metabolism both directly and also indirectly through calcium ions.

It was hypothesized that CREB1, an important transcription factor involved in lipid metabolism^38, 39^, played a role in changing lipid deposition in response to HS intake. The 2 sites (S133 and S142) were phosphorylated to a greater degree by HS intake, so CREB1 was also imported into the regulatory network. In Fig. 10B, CREB1 was shown to directly regulate the expression of Lep, Ldlr and Hmgcr, and CREB1 was, in part, regulated by Agt and Egf, both of which involved the RAS. There was evidence that calcium and genes such as Camk2a and Prkcq directly regulate CREB1. It can be deduced that CREB1 is important for regulating lipid metabolism, by mediating downstream events from genes in the RAS pathway and in calcium in response to HS intake.

## Conclusions

In this study, it is concluded that HS intake reduced abdominal fat deposition through the RAS, calcium signaling, and CREB1 in mouse adipose tissue, and an underlying molecular regulatory network mediated by calcium was established that provided a link to depressed feed intake.

## Methods

### Animals and Tissue Collection

Thirty 6-wk old female C57BL/6 mice of similar weight (±0.5 g) were purchased from Vital River (Beijing, China). All animal experiments were performed in accordance with protocols approved by

the Animal Research Committee in the Institute of Basic Medical Sciences, Chinese Academy of Medical Sciences (protocol number: ACUC-A02-2013-030). Mice were housed under a 12/12-h light/dark cycle in a SPF environment. Mice were ad libitum fed either a normal-salt diet (NS, 0.4% NaCl) or high-salt diet (HS, 96%NS + 4%NaCl). A pair-fed (PF) treatment involved providing mice with the NS diet but at the level corresponding to the previous day’s consumption by mice given the HS diet. Water was freely available throughout the experiment. After 8 wk, feed was withheld for 18 h, mice were killed and abdominal fat (AF) was obtained, live weight and fat mass were recorded, and the AFP (%) was calculated (mass of AF as a percentage of live weight). AF samples were stored at −80 °C.

### Feed intake and whole body metabolism

After feeding for 8 wk, mice from HS and NS treatments (n = 10, each) were transferred to individual cages without bedding in metabolism cages (CLAMS, comprehensive laboratory animal monitoring system, Columbus Instruments International Corp, Columbus, OH) for an additional 2 d on a 12/12 h light/dark cycle. Feed intake and metabolic indices (heat production, Heat; rate of oxygen consumption, VO_2_; and the respiratory exchange ratio, RER) were measured.

### Cell culture and NaCl treatment

The 3T3-L1 cell line was used to analyze cell differentiation. Cells were cultured in 48-well plates and treated with 0, 0.5, 5, 50, or 500 mM NaCl supplement using a classic cocktail method^40^. Ten days after starting differentiation, cells were fixed in 4% paraformaldehyde for 30 min. Cells were washed twice with PBS and immersed in a 0.3% Oil Red O solution in PBS for 2 hours. After thoroughly washing with PBS, the Oil Red O was eluted with 100% isopropanol and quantified on a microplate reader at 510 nm. Each treatment was applied to 6 replicate wells.

### Analysis of digital gene expression profile, differentially expressed genes and bioinformatics

Three abdominal fat samples from each of HS or NS mice were examined. Total RNA was isolated using a commercial kit according to the manufacturer’s protocol (DP419, Tiangen, Beijing, China). After removal of genomic DNA, RNA was used to determine the digital gene expression profile (RiboBio, Guangzhou, China). Screening of differentially expressed genes (DEGs) was performed, and genes were considered to be DEGs with FDR <0.01 and a fold change ≥1.5. Gene Ontology enrichment analysis was performed using the GOEAST software toolkit. The significance level of GO term enrichment was set as FDR-adjusted p-value less than 0.05 by the Yekutieli method. Enriched KEGG pathways with DEGs were identified by a hypergeometric test using R packages (p < 0.01, FDR adjusted). Pathways with <3 known genes were discarded. Interactions between GO, KEGG, and other pathways were performed using IPA software.

### Q-PCR and correlation analysis

To confirm results of digital gene expression profiling, the transcript abundance of 16 representative genes related to lipid metabolism were performed by q-PCR in the 2 treatments. The RNA was reverse-transcribed and subjected to q-PCR. Primer information is listed in Additional file 1. The PCR mixture contained 10 μl of 2 × iQ™ SYBR Green Supermix, 0.5 μl (10 mM) of each primer and 1 μl of cDNA, with ddH_2_O to 20 μl. Samples were amplified in the real-time PCR Detection System ABI 7500 (Applied Biosystems, Shanghai, China). A melting curve was performed to verify that only a single PCR product was amplified. Samples were assayed in triplicate with standard deviations of CT values not exceeding 0.5 on a within-run basis. Correlations between relative abundances from qPCR and digital profiling data were calculated.

### Western blotting

Abdominal fat from mice fed HS or NS diets, 3 each were used for Western blotting. Total protein levels were measured using the BCA Protein Assay Kit. The following monoclonal and polyclonal antibodies were purchased from Abcam (USA): anti-actin, anti-CREB1, anti-pCREB1 (S133), anti-pCREB1 (S142) and ATP2a1.

### ELISA

Six mice each from HS and NS diets were used. Concentrations of AGT and ANGPTL4 were measured in abdominal fat tissue using mouse-specific ELISA kits (R&D Systems, Minneapolis, MN). Abdominal fat samples were homogenized, and centrifuged at 1500 g for 20 min at 4 °C. The supernatants were immediately frozen at −80 °C until assays were performed according to the manufacturer’s instructions.

### Measurement of serum concentration of Na^+^

Concentration of Na^+^ was measured in serum of 6 mice in each of the HS and NS diets using a kit from RongSheng Biotech Co., LTD (Shanghai, China).

### Statistical Analyses

Statistical differences between the diets were evaluated using the Student’s t-test. P < 0.05 or <0.01 was considered significant. Data are presented as mean ± SEM.

## Electronic supplementary material

Supplementary file
