# Supplementary material for: High-salt intake negatively regulates fat deposition in mouse
Source: Sci Rep. 2017 May 17;7:2053. doi: 10.1038/s41598-017-01560-3 (PMC5435674; doi:10.1038/s41598-017-01560-3)
Supplement: Supplementary file 1 — Supplementary file [file 41598_2017_1560_MOESM1_ESM.pdf]

Title-High salt intake negatively regulates fat deposition in mouseWen

Huanxian Cui[#,1,2], Shuyan Yang[#,3], Maiqing Zheng[1,2], Ranran Liu[1,2], Guiping Zhao[*,1,2], Jie Wen[*,1, 2]

[1]Institute of Animal Sciences, Chinese Academy of Agricultural Sciences, Beijing 100193, China

[2]State Key Laboratory of Animal Nutrition, Beijing 100193, China

[3]Institute of Zoology, Chinese Academy of Sciences, Beijing 100101, China

[#]Huanxian Cui and Shuyan Yang contributed equally to this work.

*Corresponding author, Tel: 86-10-62815856, Fax: 86-10-62895351

E-mail: jiewen@iascaas.net.cn

Additional file 1-The specific primers for q-PCR in this study

Gene	Sequence		Accession NO.	Length(bp)
	F(5'-3')	R(5'-3')		
Leptin	CAGCTGCAAGGTGCAAGAAG	GATACCGACTGCGTGTGTGA	NM_008493	196
Fabp3	TCACTCATGGCAGTGTGGTG	ATTGACCTTGGAGCACCCTT	NM_010174	266
Cpt1b	TGTCTACCTCCGAAGCAGGA	CGGCTTGATCTCTTCACGGT	NM_009948	163
Acot3	AGCTCTTGACCTTGTCTGTCTG	GGGAGTTGGTGTTTTCCAGC	NM_134246	130
PCK1	TGCGGATCATGACTCGGATG	AGGCCCAGTTGTTGACCAAA	NM_011044	126
Pdk4	GAGCTGTTCTCCCGCTACAG	CGGTCAGGCAGGATGTCAAT	NM_013743	161
Thrsp	CGCTATCCCAAGAACTGCCT	CCTCCGTGGCAGTAGACAAC	NM_009381	278
Hmgcr	ACGATCCTTCCTTATTGGCGG	CTCCGGATCTCAATGGAGGC	NM_008255	125
Ldlr	GTGTTTGCAGCGGGAACATT	TTCTGCAACTCCGGCAGC	NM_00125265	133
Fabp5	CGAGAGCACAGTGAAGACGA	TCATGACACACTCCACGATCA	NM_010634	200
Dgat2	GGCTACGTTGGCTGGTAACT	CTTCAGGGTGACTGCGTTCT	NM_026384	196
Plin2	GCTGGAGCCAAGGATTCTGT	TCATGAACTGCACCATCCCC	NM_007408	142
Mogat2	TACAGACCTTCGCGGTCCTT	TCCATATGGCCAAGCGTCTG	NM_177448	198
Scd2	CCACCTCTACGGATATCGCC	GAACTGCAAGACCCCACAC	NM_009128	296
Acsl6	GCCTGATGACCTCTCCATCG	CCTCCGTGGCAGTAGACAAC	NM_144823	219
Agt	AGGTTGGCGCTGAAGGATAC	GATGTATACGCGGTCCCCAG	NM_007428	132
Egf	GCTCCGTCCGTCTTATCAGG	TGAGAAGTTCGGGGTCAGGA	NM_010113	249
Camk2a	TTGACGAGGGAGTAGACAGTGGA	GAACGCTGGAACTGGACTTTC	NM_009792	247
Camk2b	ACGGAATTTCTCAGCCAAGAGT	ATCCTTTGGGGCTGGTGATG	NM_00117405	104
Angptl4	AGCTCATTGGCTTGACTCCC	GAAGTCCACAGAGCCGTTCA	NM_020581	203
Pla2g4e	AATCGGCTCCAGCTCTATGC	CCCCTCCTGAGAACTGCAAG	NM_177845	244

Additional file 2-The total DEGs (HS vs NS)

Gene	FoldChange	Log2FoldChange	P-val	P-adj
1-Mar	0.449107501	-1.154867278	0.000863627	0.027270895
0610005C13Rik	3.275586106	1.711753074	0.00123264	0.036710045
1190005I06Rik	1.75579398	0.812123573	4.80E-06	0.000318117
1500012F01Rik	1.513530483	0.597917732	0.001646227	0.046172864
1810055G02Rik	0.611058811	-0.710616856	0.000254152	0.010048623
2010016I18Rik	2.091342705	1.064429494	3.18E-06	0.000220313
2010111I01Rik	0.604057131	-0.727243091	5.17E-05	0.002560736
2310015D24Rik	4.80604931	2.264851451	0.000784428	0.024998942
2310050B05Rik	3.95056342	1.982058422	0.001805724	0.049902672
2410006H16Rik	1.893454418	0.921020691	5.43E-06	0.000356373
2610016A17Rik	0.597104406	-0.74394488	0.000392593	0.014236228
2610020H08Rik	1.683343447	0.751329555	0.00114942	0.034890463
2810013P06Rik	1.630592125	0.705395953	0.000438162	0.01558828
3010033K07Rik	0.508177673	-0.976595104	3.55E-05	0.001874603
3110062M04Rik	1.513329892	0.597726516	0.000101538	0.004582586
3425401B19Rik	45.92874785	5.521325547	4.56E-40	6.61E-37
4930404N11Rik	0.619714912	-0.69032341	1.34E-05	0.000793271
4930412C18Rik	2.229055898	1.156432796	0.001232572	0.036710045
4930471I20Rik	0.440314906	-1.183392409	2.11E-05	0.001188867
4933422H20Rik	0	#NAME?	7.47E-06	0.000468512
6330416G13Rik	0.498249136	-1.005060791	9.80E-10	1.37E-07
9230116N13Rik	2.590896774	1.373451537	0.001003399	0.030856171
9330175E14Rik	2.360227913	1.238926178	0.000626892	0.020911176
9530026P05Rik	2.793907582	1.482284299	3.58E-06	0.000244264
A530016L24Rik	0.595238437	-0.748460406	6.23E-06	0.000401263
A630019I02Rik	12.68169499	3.664675679	1.40E-11	2.53E-09
A930016O22Ri	74.89540581	6.226805319	2.06E-12	4.08E-10
Aacs	0.649238822	-0.623178825	0.000203998	0.008328091
Abra	6.014346277	2.588407934	1.49E-09	2.00E-07
Acaa1b	1.703642063	0.768622256	1.83E-07	1.68E-05
Accs	1.639095493	0.712899908	0.001325089	0.039210954
Accsl	6.544139824	2.710203574	0.00020862	0.00846168
Acot3	3.245579893	1.69847627	0.000320011	0.012045231
Acsl3	0.470225673	-1.088574787	4.41E-10	6.44E-08
Acsl6	4.403660657	2.1387033	0.001764891	0.048845967
Acsm3	2.160677946	1.111484051	9.64E-09	1.13E-06
Acsm5	1.9362304	0.953250635	4.62E-08	4.85E-06
Acss2	0.427374869	-1.226426019	1.89E-05	0.00108027
Acss3	1.537650869	0.620727969	0.000201586	0.0082475
Acta1	35.77756326	5.160983226	2.53E-29	2.80E-26
Actn2	28.16717385	4.815942913	4.90E-22	3.42E-19
Actn3	50.74742164	5.665262619	1.61E-11	2.89E-09
Acvr1c	1.758598436	0.814426091	1.51E-08	1.73E-06
Adap1	0.314397841	-1.669336783	2.83E-19	1.30E-16
Adck3	2.643407188	1.402398673	6.26E-21	3.80E-18
Adssl1	3.290727719	1.71840666	4.19E-16	1.29E-13
Adtrp	2.147769326	1.102839054	9.06E-07	6.96E-05
Afap1	0.635515703	-0.654000321	0.000377523	0.013782537
Agbl1	17.30725936	4.113305384	0.000180155	0.007551248

Agpat2	0.582819807	-0.778878188	1.40E-05	0.000817994
Agt	2.41311992	1.270899612	1.60E-17	5.69E-15
Ahr	1.50805272	0.592686865	0.001314658	0.038963563
Akap5	0.57171703	-0.806626829	0.000877024	0.027601319
Alpk2	2.781374955	1.475798246	0.00080472	0.025582484
Alpk3	3.781695815	1.919033323	2.12E-12	4.16E-10
Als2cl	0.561142625	-0.83356059	5.56E-06	0.000362383
Amot	1.802215632	0.849771637	0.000762408	0.024443811
Ampd1	57.23855505	5.838915347	5.53E-08	5.71E-06
Angpt1	1.699811693	0.765374932	1.02E-05	0.000624476
Angptl4	2.504627475	1.324596041	0.000499978	0.017360858
Ank1	2.452852657	1.294460574	0.001453901	0.042356696
Ankef1	1.756346011	0.812577093	3.91E-05	0.002005119
Ankrd2	24.21837963	4.598030438	3.78E-05	0.00196227
Ankrd23	4.534677567	2.180999973	1.65E-12	3.30E-10
Anln	2.098019085	1.069027802	7.27E-06	0.00045909
Ano5	4.842121807	2.275639372	1.71E-06	0.000124472
Anxa2	0.501415522	-0.995921436	8.96E-11	1.39E-08
Apln	0.509872431	-0.971791763	8.16E-07	6.37E-05
Apobec1	0.652024837	-0.617001174	0.000859642	0.027190683
Apobec2	38.23762599	5.25692105	4.04E-49	9.51E-46
Appl2	1.591375098	0.670273929	2.38E-05	0.001319568
Arhgef37	0.545373527	-0.874683423	0.000322915	0.01212557
Art1	18.98976565	4.247150196	9.93E-12	1.85E-09
Arxes2	1.597040272	0.675400693	4.34E-06	0.000291852
Asb10	4.562723114	2.189895107	6.63E-07	5.31E-05
Asb11	37.36247277	5.223518035	1.69E-14	3.98E-12
Asb14	5.583407981	2.481145978	0.001119432	0.03420083
Asb15	19.11621283	4.25672483	2.60E-07	2.26E-05
Asb16	30.63521856	4.937119239	1.57E-07	1.48E-05
Atp10a	0.577323798	-0.792547398	6.04E-06	0.000390454
Atp2a1	61.61272023	5.945156328	2.93E-18	1.17E-15
B330016D10Rik	1.749955882	0.807318551	0.001385268	0.040608647
B3galt2	1.931531456	0.949745173	1.57E-08	1.78E-06
B3gnt8	0.60538633	-0.724071995	0.00150197	0.043206971
Barx2	10.87150535	3.442479815	0.000162287	0.006878906
BC051226	1.918003495	0.939605349	1.85E-05	0.001062155
Bcat2	1.753649445	0.810360381	0.000127389	0.00557547
Bhlhe41	0.576498634	-0.794610906	0.000420207	0.015063413
Bivm	1.708305952	0.77256638	0.000408833	0.014711746
Bmp3	1.735496437	0.795348403	4.64E-05	0.002318605
Bmyc	0.47237927	-1.081982441	1.61E-08	1.81E-06
Bnip3	1.601103246	0.679066342	4.18E-06	0.000282222
Btg1	1.545702143	0.628262339	1.19E-05	0.000716916
C4b	1.608822793	0.686005426	0.00060977	0.020492613
Cables1	0.496622334	-1.009778951	0.000565273	0.019307075
Cacna1s	31.88788129	4.99493634	1.56E-20	8.91E-18
Cacng1	14.40988167	3.848986584	0.001660458	0.046502719
Cadps2	1.547314307	0.629766282	0.000160345	0.006827345
Cald1	0.653467912	-0.613811699	7.43E-05	0.003523871
Calr3	1.740947252	0.799872492	0.001118067	0.03420083
Camk2a	4.850225556	2.27805184	3.56E-05	0.001875075
Camk2b	4.439348046	2.150347821	1.25E-11	2.30E-09

Capn11	17.21370618	4.105485844	1.21E-21	7.83E-19
Capn3	4.478474739	2.163007469	1.49E-07	1.43E-05
Car4	1.536738188	0.619871396	8.36E-05	0.003925749
Casq1	19.63077632	4.295045322	2.29E-15	6.25E-13
Cav3	9.23868415	3.207687386	9.20E-09	1.08E-06
Ccl12	0.32165696	-1.63640519	9.26E-05	0.004292562
Ccl8	2.57709334	1.365744791	0.001707724	0.047613873
Ccnd1	0.552420226	-0.856161953	2.31E-07	2.05E-05
Ccng2	1.558482465	0.640141922	0.000237711	0.009458202
Cd14	1.841489535	0.880873198	0.001469077	0.042653177
Cd274	2.358142877	1.237651132	5.54E-15	1.45E-12
Cd300a	1.890108738	0.918469235	0.000223305	0.008960773
Cdkn2b	0.580250482	-0.78525228	0.000151945	0.006528797
Cecr2	2.140355831	1.097850662	1.66E-07	1.56E-05
Cela1	0.540912898	-0.886531795	0.000213199	0.008610298
Ces1d	1.568087016	0.64900562	0.000189406	0.007868902
Chrdl1	1.926759529	0.946176526	8.11E-11	1.28E-08
Chst1	1.881898217	0.912188601	9.17E-10	1.29E-07
Chsy1	0.351174248	-1.509741039	2.97E-10	4.54E-08
Ciita	1.913415016	0.936149826	4.57E-06	0.000304879
Cisd3	0.660131274	-0.599175148	6.17E-05	0.003015788
Ckm	76.5213037	6.257789548	1.33E-14	3.24E-12
Ckmt2	207.2343425	7.695119293	1.94E-32	2.44E-29
Clcn1	9.760283212	3.286923011	7.92E-08	7.85E-06
Clec10a	1.51128237	0.595773241	0.001141408	0.034703241
Clmn	1.50518847	0.589944143	4.38E-05	0.002212378
Cmklr1	0.48129197	-1.055015742	2.02E-09	2.62E-07
Cmya5	13.25050751	3.727975712	1.57E-14	3.75E-12
Cntnap2	23.62417515	4.562192053	0.000279073	0.010784728
Col15a1	0.589368266	-0.762758712	7.97E-07	6.25E-05
Col5a3	0.511827039	-0.96627173	3.95E-11	6.58E-09
Coq10b	0.636998366	-0.650638424	0.000457561	0.016156274
Cox6a2	33.63158388	5.071744819	6.88E-09	8.20E-07
Cpt1b	1.851251718	0.888501075	0.000295461	0.011301971
Crebrf	1.971857787	0.979555506	3.12E-10	4.66E-08
Csf2rb2	1.808397092	0.854711503	0.000323434	0.01212557
Cspg4	0.649302432	-0.623037481	1.68E-05	0.000970253
Csrp3	34.64490997	5.114571502	3.52E-20	1.95E-17
Cyp2e1	3.245771191	1.698561301	1.17E-17	4.22E-15
Cyp2f2	2.207178739	1.142203465	1.68E-08	1.87E-06
D4Wsu53e	1.524661044	0.608488545	7.43E-05	0.003523871
D630039A03Ri	4.959398677	2.310165206	5.17E-07	4.25E-05
D830015G02Ri	11.48932727	3.522222422	5.85E-08	5.97E-06
Dctd	0.491698599	-1.024153851	0.000618977	0.020764965
Ddit4l	2.963839816	1.567467478	5.06E-07	4.17E-05
Dfna5	0.453149977	-1.141939483	6.44E-08	6.48E-06
Dgat2	0.488206844	-1.034435576	6.29E-07	5.10E-05
Dhcr24	0.667410904	-0.583352838	9.03E-05	0.004208378
Dhcr7	0.510154947	-0.970992597	0.001535019	0.043885418
Dhrs7	0.442215314	-1.177179109	2.91E-07	2.51E-05
Dhrs7c	66.8990499	6.063913817	3.58E-11	6.01E-09
Dhx32	0.519152337	-0.945770158	5.87E-08	5.97E-06
Disp2	0.403179305	-1.310506507	9.83E-05	0.004467461

Dmrt2	0.579422905	-0.787311379	3.41E-06	0.000233064
Dnaja1	0.661062417	-0.597141598	0.000566286	0.019307075
Dnmt3l	0.199588401	-2.324900216	1.21E-09	1.68E-07
Dock9	1.532851327	0.616217776	2.54E-05	0.001392461
Dram1	1.511564788	0.596042816	3.11E-05	0.001676447
Dusp13	4.751426459	2.2483607	0.000723719	0.023362596
Dusp27	5.624473975	2.491718176	2.07E-07	1.87E-05
Dusp8	0.467002166	-1.098498855	1.48E-06	0.000109733
E130102H24Rik	1.896079202	0.923019229	0.000167972	0.007087986
E130309D14Rik	2.113058357	1.079332611	0.001362853	0.040240547
E2f2	0.452185445	-1.145013541	2.07E-08	2.28E-06
Eef1a2	25.38708342	4.666022756	5.63E-19	2.52E-16
Egf	3.06089373	1.613952957	1.99E-06	0.000142172
Elmo3	0.581681742	-0.781698074	3.61E-05	0.001895517
Eno3	6.321589633	2.660287386	8.08E-55	2.17E-51
Epb4.1l2	0.622138177	-0.684693057	9.71E-06	0.000597186
Epb4.9	0.613080126	-0.705852457	0.000110817	0.004977495
Ephx2	1.528657333	0.612265045	2.61E-05	0.001417061
Eri2	2.139967305	1.097588755	1.35E-05	0.000794659
Esm1	0.414080285	-1.27201758	1.46E-05	0.000847956
Exd1	1.933564782	0.951263101	4.61E-05	0.002309539
Fabp3	6.489993857	2.698217113	5.26E-13	1.09E-10
Fabp5	0.542316324	-0.882793499	2.25E-08	2.46E-06
Fam134b	2.672078785	1.417962546	6.51E-09	7.80E-07
Fam210b	1.61053625	0.687541133	6.51E-06	0.000418237
Fam26e	0.543722398	-0.879057835	0.001214831	0.036348363
Fam50b	0.082434913	-3.600600718	5.68E-08	5.85E-06
Fam69b	0.373289379	-1.421633635	3.53E-14	8.19E-12
Fbp2	6.397738568	2.677562041	5.34E-09	6.48E-07
Fbxo21	1.823609357	0.866796717	4.47E-09	5.53E-07
Fbxo31	1.666370919	0.736709567	6.96E-07	5.53E-05
Fbxo40	74.6881784	6.222808007	5.79E-14	1.31E-11
Fbxw17	0.567497222	-0.817314765	0.000881205	0.027658753
Fcamr	3.663102914	1.873066234	0.000699834	0.022826476
Ffar2	1.988433996	0.991632675	1.22E-09	1.68E-07
Fgfr3	0.520503054	-0.942021465	1.13E-05	0.000682185
Fign	1.679806316	0.748294898	0.00152066	0.043626245
Fitm1	43.51412923	5.443412021	1.07E-15	3.09E-13
Fosl2	0.650719188	-0.619893	2.57E-05	0.001402911
Frat1	2.059584137	1.042353064	1.76E-06	0.000126712
Frat2	3.789422964	1.921978178	2.45E-09	3.13E-07
Fst	0.617103191	-0.696416341	0.000673047	0.022105996
Fstl3	0.553248993	-0.853999174	0.000125322	0.005508459
G6pdx	0.603804673	-0.727846172	6.83E-06	0.000436023
Gale	0.651586553	-0.617971264	0.001389613	0.040672644
Galnt2	0.617217489	-0.696149153	2.94E-06	0.000204679
Gbp10	3.498645523	1.806796501	0.001584038	0.044964707
Gbp11	2.580048086	1.367397954	0.000198308	0.008148823
Gbp4	2.981989323	1.576275092	2.10E-25	2.08E-22
Gbp5	1.919198758	0.940504129	1.77E-07	1.64E-05
Gbp7	1.812789653	0.858211532	4.43E-09	5.53E-07
Gbp9	1.785584671	0.836396547	8.91E-08	8.73E-06
Gimap4	1.527737428	0.611396609	0.000309357	0.011767859

Gm12185	3.218995114	1.686610387	0.001367802	0.040284868
Gm12250	2.559193017	1.355688961	5.47E-11	8.79E-09
Gm4070	1.990268376	0.992962983	0.001671314	0.046667861
Gm4285	1.848031186	0.885989103	0.000332246	0.012406496
Gm4814	51.81190633	5.695211761	2.40E-11	4.14E-09
Gm4951	2.026053475	1.018672253	6.65E-07	5.31E-05
Gm5177	0.613220526	-0.705522107	3.27E-05	0.001751169
Gm6277	0.459613999	-1.121505354	4.93E-07	4.12E-05
Gm9992	5.423180127	2.439139089	1.89E-08	2.10E-06
Golga7b	0.431681991	-1.211959187	0.000655637	0.021609628
Gp1ba	2.08288727	1.05858476	0.000963793	0.02968671
Gpr182	1.811706081	0.857348922	0.000115645	0.005146527
Gpr81	1.816839609	0.861431063	2.01E-09	2.62E-07
Gprin2	0.350963837	-1.51060571	0.001439763	0.042009822
Gpt	0.654194503	-0.612208459	3.29E-05	0.001758655
Gramd2	0.138720543	-2.849746644	2.28E-06	0.000161255
Grb7	0.341620812	-1.549532226	6.10E-08	6.17E-06
Grhl1	2.004063466	1.002928198	4.33E-05	0.002196846
Gss	0.560968559	-0.834008182	2.34E-07	2.06E-05
Gulp1	1.652140921	0.724336749	0.000269903	0.010561045
Gvin1	1.990268376	0.992962983	0.001671314	0.046667861
Gzma	6.095902678	2.60783987	0.000111917	0.005009636
H19	8.417830874	3.073448524	7.48E-30	8.80E-27
H2-K2	0.57471963	-0.799069768	1.08E-05	0.000655229
H2-Q6	1.778699604	0.830822881	0.000956416	0.029556227
H2-Q8	1.765750157	0.820281225	0.001380068	0.040569718
Hephl1	0.286887859	-1.80144118	9.88E-06	0.000605383
Hey1	0.60764391	-0.718701969	0.000271193	0.010561045
Hfe2	4.472142911	2.160966292	3.89E-09	4.91E-07
Hhatl	7.263239489	2.86061315	1.10E-08	1.27E-06
Hk2	0.477435966	-1.066620844	1.92E-05	0.001094309
Hkdc1	0.048988045	-4.351426469	4.38E-05	0.002212378
Hmgcr	0.490607258	-1.027359518	3.13E-09	3.98E-07
Hoxb6	0.653872341	-0.612919096	0.001176727	0.035490397
Hp	1.562198444	0.643577728	0.000305833	0.011674992
Hr	0.568081278	-0.815830737	1.85E-07	1.69E-05
Hrc	13.24996283	3.727916407	2.81E-45	5.30E-42
Hs3st3b1	1.751612745	0.808683852	5.20E-05	0.002569894
Hs6st2	7.615230623	2.928887729	0.000317724	0.011983098
Hspa12a	0.660529487	-0.598305128	4.12E-05	0.002103422
Hspb6	2.148948711	1.103631051	1.56E-09	2.08E-07
Hspb8	0.64967741	-0.622204553	0.000732097	0.023592577
Hvcn1	0.451511952	-1.147163917	4.34E-07	3.66E-05
I830012O16Rik	0.565674492	-0.821955977	0.00064061	0.02126328
Ifi47	2.04531157	1.032320631	6.21E-09	7.49E-07
Ifit3	0.559834572	-0.836927513	7.31E-07	5.76E-05
Igfn1	15.92747598	3.993445757	8.85E-07	6.82E-05
Igsf8	0.600475055	-0.73582378	5.09E-06	0.000335051
Igtp	2.478583578	1.309515907	5.94E-18	2.24E-15
Iigp1	2.541346565	1.34559313	1.07E-06	8.18E-05
Il22ra2	8.489467428	3.085674052	0.000119205	0.005278664
Inca1	1.54939483	0.631704831	0.001715718	0.047765983
Insig1	0.487810662	-1.035606804	5.60E-10	8.05E-08

Irf1	1.674240375	0.743506674	6.66E-07	5.31E-05
Irgm1	1.516294056	0.600549564	0.000132846	0.00577894
Irgm2	2.054560557	1.038829854	2.28E-07	2.03E-05
Itgb1bp2	6.135697666	2.617227396	4.75E-06	0.000315717
Itgb6	9.288415636	3.215432531	5.05E-07	4.17E-05
Ivns1abp	1.977855223	0.983936826	1.30E-11	2.37E-09
Jakmip3	0.259260847	-1.947523746	5.76E-07	4.69E-05
Jph1	2.243726798	1.16589702	0.000157747	0.006747267
Jsrp1	16.11230784	4.010091248	8.93E-24	7.64E-21
Kcna7	11.08628997	3.470704742	5.40E-17	1.85E-14
Kcnb1	0.550404463	-0.861435926	8.65E-07	6.72E-05
Kcnc1	5.069325209	2.341793719	0.000350821	0.012996941
Kcnc4	2.902571169	1.537331441	0.000132959	0.00577894
Kcng4	0.353080818	-1.501929652	7.30E-06	0.0004596
Kcnj11	8.665119074	3.115219575	2.29E-12	4.45E-10
Kcnj12	2.818000212	1.49467172	0.000199634	0.008185449
Klf11	1.908750634	0.932628636	4.54E-05	0.002277981
Klhl21	1.745989496	0.80404488	1.72E-07	1.60E-05
Klhl24	1.993890442	0.995586141	2.07E-11	3.67E-09
Klhl30	3.31054239	1.727067604	7.56E-05	0.003575126
Klhl31	42.3307672	5.403634731	2.71E-11	4.63E-09
Klhl33	2.213031874	1.14602423	0.000522353	0.018028652
Klhl40	27.91325032	4.802878222	3.65E-10	5.36E-08
Klhl41	26.28809001	4.71633742	5.12E-26	5.36E-23
Kntc1	0.045402896	-4.461071876	7.13E-05	0.003408016
Ky	4.666018078	2.222191897	1.34E-13	2.87E-11
Lacc1	0.51797335	-0.949050222	1.06E-07	1.04E-05
Ldb3	5.313123356	2.409560209	3.01E-40	4.72E-37
Ldlr	0.398120594	-1.328722594	1.75E-19	8.22E-17
Leo1	0.61981306	-0.690094941	5.23E-05	0.002574411
Lep	0.411466132	-1.281154407	1.55E-16	5.03E-14
Lgals3	0.638189667	-0.647942846	0.000189215	0.007868902
Lgals4	1.658414412	0.729804559	7.58E-05	0.003576095
Lgals6	1.602355348	0.680194124	0.000597797	0.020126193
Lmod2	52.43485781	5.712454305	4.05E-24	3.81E-21
Lmod3	6.491170454	2.698478641	7.24E-14	1.58E-11
Lpgat1	0.613851664	-0.704038022	4.24E-05	0.002158143
Lrrc2	3.824589931	1.935305072	9.06E-06	0.000559174
Lrrc27	0.585751035	-0.771640498	3.85E-05	0.001983404
Lrrc38	23.05642006	4.52709662	6.34E-05	0.003085493
Lrrc39	0.658820397	-0.602042873	0.00022548	0.009028805
Map6	0.621281875	-0.686680129	4.06E-06	0.000275009
Mapk8ip1	0.617920807	-0.694506141	3.75E-05	0.001956573
Mapkapk3	0.554860822	-0.849802155	2.99E-08	3.22E-06
Mb	6.652456123	2.73388709	9.26E-46	1.94E-42
Mef2c	1.598848519	0.677033259	3.40E-05	0.001801594
Mettl7a1	1.508259019	0.592884209	6.58E-05	0.003181693
Mfsd12	0.61363729	-0.704541939	2.96E-06	0.000205708
Mgst3	1.546721736	0.629213671	1.40E-05	0.000817994
Mid1ip1	0.354740915	-1.495162358	2.16E-11	3.79E-09
Mir1901	0.396136973	-1.335928736	2.33E-05	0.001300434
Mlf1	3.280388208	1.713866556	1.85E-06	0.000133029
Mlip	6.216675609	2.6361433	3.62E-05	0.001898023

Mlx	0.577334418	-0.79252086	8.88E-06	0.00054947
Mogat2	0.408532907	-1.291475804	1.55E-12	3.14E-10
Mpeg1	1.985734846	0.989672993	1.62E-09	2.13E-07
Mrap	0.515106395	-0.957057644	4.78E-05	0.002377867
Msh5	3.296746846	1.721043108	0.001285985	0.038173884
Mss51	5.875740196	2.554770607	0.000377885	0.013782537
Mtfp1	0.608362835	-0.716996074	3.15E-05	0.001694875
Mtss1	0.645462289	-0.631595286	2.38E-05	0.001319568
Murc	11.28513304	3.496351521	2.90E-08	3.14E-06
Mvd	0.354129602	-1.49765065	1.09E-07	1.06E-05
Mvk	0.578143584	-0.79050026	1.04E-05	0.000633019
Myadml2	4.118700418	2.042189193	0.000115674	0.005146527
Mybpc1	11.71648236	3.550467589	5.56E-69	2.09E-65
Mybpc2	75.90212853	6.246068439	5.16E-20	2.63E-17
Mybph	1.773787625	0.826833286	3.93E-06	0.000267212
Mycl1	4.944780553	2.305906496	5.76E-21	3.61E-18
Myf6	61.04008047	5.931684959	6.10E-05	0.002988324
Myh1	33.8342002	5.08041038	5.83E-159	1.10E-154
Myh2	26.95474783	4.752467508	8.30E-21	4.88E-18
Myh4	110.5217934	6.788187067	6.49E-15	1.67E-12
Myh7	15.9706915	3.997354875	1.66E-44	2.83E-41
Myl1	2.732810156	1.450385241	1.07E-14	2.65E-12
Myl2	10.2729991	3.360785518	2.35E-05	0.001306568
Mylk2	12.85079122	3.683785283	1.14E-13	2.46E-11
Mylk4	19.5421513	4.28851739	1.23E-19	5.93E-17
Mylpf	36.92063239	5.206355359	8.17E-16	2.40E-13
Myo18b	13.47366356	3.752070276	4.31E-09	5.40E-07
Myog	25.27324155	4.659538811	0.00160102	0.045378319
Myom2	59.70765173	5.899843924	5.95E-62	1.86E-58
Myot	68.52306097	6.098517694	6.94E-18	2.56E-15
Myoz1	65.10954327	6.024797113	6.03E-10	8.59E-08
Myoz2	36.0059104	5.170161841	2.53E-18	1.04E-15
Myoz3	16.36990247	4.032973821	3.70E-18	1.45E-15
Mypn	49.38441026	5.625983777	1.06E-18	4.52E-16
N4bp2l1	1.821796487	0.865361805	1.16E-06	8.76E-05
Ncan	0.401207839	-1.317578299	3.27E-06	0.000224896
Nctc1	50.23757794	5.650695005	1.35E-16	4.55E-14
Neb	26.57163363	4.731815023	5.12E-20	2.63E-17
Net1	1.941421582	0.957113435	5.08E-11	8.32E-09
Neu2	13.59760673	3.765280845	0.000443519	0.015719432
Neurl1a	2.543523341	1.346828334	1.53E-06	0.000112694
Nfkbil1	1.580939317	0.660781992	0.000267964	0.010528339
Nlrc5	1.689394283	0.756506074	5.73E-07	4.69E-05
Nol3	0.578048512	-0.790737521	3.65E-07	3.11E-05
Npas4	0.440058335	-1.184233313	0.000338456	0.012613361
Nr1d1	1.931803818	0.94994859	0.001203146	0.036113582
Nrap	34.6188614	5.113486371	5.92E-91	2.80E-87
Nrip1	1.578803694	0.6588318	6.53E-05	0.003168659
Nt5e	3.441658706	1.783104039	1.71E-07	1.60E-05
Ntsr2	0.465774809	-1.102295479	1.40E-06	0.00010397
Nupr1l	2.964330481	1.567706297	6.99E-05	0.003345477
Nxpe5	2.075141628	1.053209804	8.72E-08	8.60E-06
Obscn	39.67271926	5.310075382	3.90E-123	3.67E-119

Odf3l1	0.539575933	-0.890102094	6.28E-05	0.003061271
Olfm1	0.613006382	-0.706026002	1.38E-06	0.000103045
Olfr56	2.667316854	1.415389215	0.000292971	0.011252471
Ophn1	1.531597522	0.615037231	9.24E-05	0.004292562
Osm	0.17556744	-2.509902785	0.001169594	0.035331868
Palm2	0.472742196	-1.080874453	1.52E-08	1.74E-06
Paqr7	0.643541395	-0.635895144	0.000361026	0.013348757
Paqr9	0.547646367	-0.868683495	0.000177745	0.007466893
Pard6g	1.548254285	0.630642439	0.000473454	0.01662388
Pbld1	1.772303595	0.825625758	0.001759218	0.048760664
Pck1	5.352804386	2.420294932	4.68E-13	9.79E-11
Pde4dip	2.546994355	1.348795762	3.37E-12	6.40E-10
Pdk4	2.481568434	1.31125224	1.82E-15	5.04E-13
Pdlim7	0.663058134	-0.592792729	0.000100947	0.004566878
Peg3	3.202765264	1.679318064	2.09E-09	2.70E-07
Peg3as	3.602845499	1.849136786	0.000745935	0.023997436
Pfkfb4	0.555145512	-0.849062122	2.46E-06	0.000173099
Pfkm	2.209119342	1.143471359	5.46E-14	1.25E-11
Pgam2	17.32753414	4.114994455	1.00E-14	2.51E-12
Pgbd5	0.446267288	-1.164020036	0.000708669	0.022955501
Pgm3	0.33278259	-1.587348136	7.06E-06	0.000447066
Phlda3	0.551780291	-0.85783417	6.45E-07	5.21E-05
Pigl	1.776776527	0.829262239	3.82E-05	0.001973203
Pim3	1.995325356	0.99662401	2.36E-11	4.12E-09
Pinx1	0.538292461	-0.893537876	0.000309516	0.011767859
Pla2g4e	23.13139198	4.531780181	8.17E-06	0.000511078
Plin2	0.474884254	-1.074352175	2.64E-05	0.001427753
Pmp22	0.589095086	-0.763427576	2.14E-07	1.93E-05
Pmvk	0.518691741	-0.947050696	2.72E-07	2.36E-05
Pnpla3	0.561840469	-0.831767551	1.71E-06	0.000124409
Pnrc1	1.964412506	0.974097912	3.03E-10	4.56E-08
Podnl1	0.390282545	-1.357409153	0.000519092	0.017958294
Polr3g	0.578286067	-0.790144753	4.09E-05	0.002090499
Popdc3	4.841595245	2.275482476	0.000785036	0.024998942
Ppa1	1.644865503	0.717969623	1.01E-06	7.71E-05
Ppan	0.655268452	-0.60984202	0.001364159	0.040240547
Ppcs	0.657047826	-0.605929708	0.000270066	0.010561045
Ppm1k	1.691158592	0.758011958	3.78E-05	0.00196227
Ppp1r14c	35.8094063	5.162266695	1.50E-12	3.07E-10
Ppp1r27	72.56758148	6.181253284	1.10E-08	1.27E-06
Ppp1r3a	33.84142622	5.080718466	2.50E-07	2.20E-05
Ppp1r3b	0.282726959	-1.822518638	1.50E-10	2.32E-08
Ppp2r5b	0.387383914	-1.368164049	3.31E-17	1.15E-14
Prkcdbp	0.567950459	-0.816163002	2.61E-08	2.84E-06
Prkcq	3.399059251	1.765135511	3.25E-07	2.78E-05
Prob1	1.954217147	0.966590784	0.000363894	0.013402115
Prr5l	1.841904344	0.88119814	9.31E-05	0.004299343
Prrt4	0.275020346	-1.862389739	5.89E-14	1.32E-11
Prss35	0.387028958	-1.369486579	0.000314355	0.011903763
Prss57	0.539807278	-0.889483666	0.000427929	0.015311058
Ptch2	0.546433891	-0.871881129	9.68E-05	0.004434659
Pvalb	112.1819827	6.809697176	3.04E-12	5.84E-10
Pygm	7.351196527	2.877979091	1.12E-21	7.52E-19

Rab32	0.52811147	-0.92108562	4.79E-09	5.85E-07
Rad51d	1.514662615	0.598996475	0.000133741	0.00579954
Raf1	0.608985941	-0.715519173	8.81E-07	6.82E-05
Ralgds	1.516065588	0.600332169	9.82E-05	0.004467461
Rasd2	1.993683046	0.99543607	0.000154395	0.006618919
Rassf3	0.503212709	-0.990759735	1.34E-05	0.000793271
Rassf6	2.53330554	1.34102109	4.48E-05	0.002252233
Rbfox1	15.39450743	3.944343802	2.55E-07	2.23E-05
Rbm24	2.709218186	1.437876585	1.59E-06	0.00011708
Rcor2	0.410497707	-1.28455393	5.05E-07	4.17E-05
Retsat	1.732528766	0.792879306	7.63E-08	7.60E-06
Rhbdl3	0.600671264	-0.735352448	2.43E-05	0.001343524
Rhob	0.551368531	-0.858911167	5.26E-08	5.47E-06
Rpia	0.568744775	-0.814146709	1.05E-05	0.00063913
Rpl3l	48.26594731	5.59293379	1.50E-22	1.18E-19
Rps6ka5	1.718205669	0.780902737	0.000663279	0.021823265
Rragd	6.730592358	2.750733482	1.78E-07	1.64E-05
Rtp4	0.656054102	-0.608113303	0.000245813	0.009739359
Rufy4	1.786154141	0.836856587	0.00150375	0.043206971
Rwdd2a	0.227097918	-2.138613613	4.86E-15	1.29E-12
Ryr1	35.24234969	5.13923821	3.73E-20	2.01E-17
Sbk1	0.429500835	-1.21926716	1.50E-07	1.43E-05
Scd2	0.423367545	-1.240017416	0.000271602	0.010561045
Scn1b	1.658694564	0.73004825	1.68E-06	0.000122823
Scn4a	19.88152714	4.313356672	4.78E-09	5.85E-07
Scn4b	3.657473142	1.87084727	3.01E-16	9.61E-14
Sctr	1.920604012	0.941560097	0.000570424	0.019377952
Sel1l3	3.296779056	1.721057203	0.000125322	0.005508459
Serinc2	6.259543017	2.646057336	0.000125565	0.005508459
Serpina3g	2.503638742	1.324026406	0.000373284	0.013667729
Serpina3k	3.638903892	1.863503948	1.34E-09	1.83E-07
Serpine1	0.402500131	-1.312938842	1.29E-07	1.24E-05
Serpinh1	0.608117401	-0.717578222	2.26E-05	0.001268418
Setd8	0.655550703	-0.609220727	3.34E-05	0.001779178
Sgca	2.998961714	1.584463104	6.97E-05	0.003345477
Sgcg	3.333581737	1.737073101	7.37E-08	7.38E-06
Sh2b2	0.610850287	-0.711109261	1.65E-05	0.000956753
Sh3rf2	3.195610215	1.676091446	9.68E-05	0.004434659
Shisa2	2.344804318	1.22946753	0.000194119	0.008011666
Shisa4	1.947517772	0.961636494	0.000403359	0.01457047
Shroom1	0.520658911	-0.941589537	0.000112065	0.005009636
Six4	2.203415345	1.139741469	0.001534045	0.043885418
Slc16a12	0.632043066	-0.66190523	0.001167076	0.035312496
Slc1a3	2.217125658	1.148690539	3.23E-15	8.69E-13
Slc22a23	1.558781165	0.640418404	1.37E-05	0.00080318
Slc25a10	0.572658995	-0.804251791	2.77E-06	0.000193906
Slc25a19	0.660256786	-0.598900872	2.57E-05	0.001402911
Slc25a25	0.659601129	-0.600334228	3.87E-05	0.001987875
Slc25a34	4.560304499	2.189130159	3.48E-11	5.90E-09
Slc26a10	1.782897013	0.83422337	0.001199181	0.036054047
Slc35g1	0.462508695	-1.112447608	1.99E-06	0.000142172
Slc39a6	0.651393317	-0.618399177	0.000923385	0.028724133
Slc8a3	3.820715446	1.933842815	5.56E-06	0.000362383

Slco2b1	1.554085889	0.636066239	2.50E-05	0.001377469
Slmo1	0.483636564	-1.048004774	4.50E-06	0.000301617
Sln	29.86835105	4.900545687	2.51E-22	1.89E-19
Smpx	65.34851371	6.030082519	3.41E-22	2.47E-19
Smtnl1	23.80431476	4.573151195	5.24E-23	4.29E-20
Smyd1	2.890194084	1.531166376	3.32E-13	7.02E-11
Snhg11	0.500961337	-0.99722883	4.25E-07	3.60E-05
Sorl1	0.612535818	-0.707133888	0.00026168	0.010324572
Spire1	0.640764969	-0.642132819	7.21E-05	0.00343535
Spry3	0.086394753	-3.532912494	0.000127873	0.005583697
Spry4	0.463151959	-1.110442479	6.66E-05	0.003214676
Sptb	3.622228119	1.856877407	8.02E-16	2.40E-13
Sqle	0.396453831	-1.334775228	0.001064402	0.032678693
Srgap3	1.8934625	0.921026849	0.000240234	0.009538415
Srl	5.348065444	2.41901712	6.64E-16	2.02E-13
Ssh2	1.716442768	0.779421754	2.96E-07	2.54E-05
St3gal6	2.199473174	1.137158006	1.30E-06	9.74E-05
Stac3	4.117719154	2.041845435	2.59E-05	0.001408652
Strip2	2.268860472	1.18196789	1.45E-09	1.97E-07
Sult1a1	1.668926473	0.738920396	0.000283564	0.010935799
Syne3	1.561067859	0.642533252	3.40E-05	0.001801594
Synpo2l	7.775001704	2.958842991	1.41E-16	4.65E-14
Sypl2	10.10970154	3.337668502	8.27E-20	4.10E-17
Tap1	1.951544977	0.964616713	7.04E-06	0.000447066
Tapt1	1.510518644	0.595043991	0.000189357	0.007868902
Tbx15	15.26576931	3.93222839	1.46E-15	4.16E-13
Tcap	36.67709286	5.196807386	5.96E-91	2.80E-87
Tcea3	1.827149586	0.86959475	0.000381958	0.013904141
Tcp11l2	1.836426664	0.876901285	9.32E-05	0.004299343
Tctn1	0.604348295	-0.72654786	1.80E-05	0.001039888
Tfap4	1.94100518	0.956803968	0.000196917	0.008109374
Tgtp2	2.454222767	1.295266207	8.28E-06	0.000515952
Thrsp	0.563493183	-0.827529937	0.001381785	0.040569718
Tlcd1	0.658446502	-0.602861868	3.67E-05	0.001916739
Tlcd2	0.367589473	-1.443832645	7.12E-07	5.63E-05
Tm7sf2	0.63338036	-0.658855963	9.62E-05	0.004427342
Tmem140	1.580765413	0.660623286	1.93E-05	0.001095524
Tmem179	0.645427151	-0.631673827	2.22E-05	0.001248896
Tmem38a	3.015912239	1.592594448	6.62E-11	1.06E-08
Tmem45b	0.503791996	-0.989099892	4.43E-11	7.31E-09
Tmem79	0.347321419	-1.525656713	1.62E-09	2.13E-07
Tmem86a	1.612644755	0.689428666	7.67E-05	0.003608527
Tmod4	24.32542643	4.604393191	4.14E-08	4.38E-06
Tnfrsf12a	0.448745967	-1.156029121	0.001470877	0.042653177
Tnfsf10	1.711408845	0.775184452	1.30E-05	0.000778701
Tnnc1	2.616477751	1.387625991	1.29E-05	0.000773074
Tnnc2	44.98500273	5.491372205	1.41E-14	3.41E-12
Tnni1	4.789102742	2.259755386	3.21E-06	0.000221244
Tnni2	64.85067014	6.019049578	3.61E-12	6.79E-10
Tnnt1	4.372952242	2.128607592	1.15E-06	8.72E-05
Tnnt3	58.90112487	5.880223281	4.81E-18	1.85E-15
Tppp	1.632060561	0.706694593	1.73E-06	0.000125221
Trabd2b	0.518107238	-0.948677355	8.62E-11	1.35E-08

Trdn	7.673392274	2.939864509	7.49E-24	6.71E-21
Trim54	38.34689413	5.261037828	9.26E-19	4.05E-16
Trim63	29.11973624	4.863925383	1.65E-18	6.89E-16
Trim72	13.19147736	3.721534241	4.09E-16	1.28E-13
Trit1	1.698366944	0.764148196	4.98E-06	0.000328753
Trp53inp2	0.578575045	-0.789423997	1.11E-07	1.07E-05
Trub2	0.450542049	-1.150266336	8.00E-15	2.04E-12
Tsku	0.539928028	-0.889160984	5.70E-06	0.000370185
Tst	1.876006924	0.907665153	8.65E-10	1.22E-07
Ttc25	0.446875299	-1.162055793	0.000193886	0.008011666
Ttn	56.77753369	5.827248277	6.86E-14	1.52E-11
Tuba1a	0.280653992	-1.833135514	4.70E-33	6.32E-30
Tuba1b	0.49777444	-1.006435944	3.23E-10	4.78E-08
Tuba8	2.055194285	1.039274783	5.15E-05	0.002555354
Tubb2a	0.568107101	-0.815765158	4.52E-07	3.79E-05
Tubb3	0.102065164	-3.292437556	0.00090067	0.02815715
Tubb4b	0.478066668	-1.064716273	4.55E-10	6.58E-08
Tubb5	0.565234697	-0.823078067	4.95E-08	5.18E-06
Tubb6	0.551241963	-0.859242379	1.58E-08	1.78E-06
Txlnb	11.33465386	3.502668429	7.60E-09	8.99E-07
Ubd	3.620204718	1.856071282	0.000120652	0.005330203
Ucp3	0.538996277	-0.891652786	2.68E-06	0.000187926
Unc119	0.636262799	-0.652305323	8.55E-06	0.000530943
Unc5a	0.626429756	-0.674775352	2.28E-06	0.000161255
Unc93a	3.695075045	1.885603665	2.02E-07	1.84E-05
Utp14b	0.444069823	-1.17114156	5.47E-05	0.002686704
Vgll2	31.5525465	4.97968454	0.000118071	0.005240775
Vnn1	2.66322743	1.413175635	0.000159319	0.006799058
Vwa3a	1.594334729	0.672954554	0.00062778	0.020911176
Wisp2	0.226471432	-2.14259902	3.02E-10	4.56E-08
Wscd2	0.599154753	-0.738999416	1.86E-05	0.001069898
Xirp1	2.347698017	1.231246847	2.04E-05	0.001155181
Xirp2	37.65623674	5.234816923	1.49E-15	4.19E-13
Yipf7	16.97915466	4.085692728	0.000881788	0.027658753
Zbtb5	1.657604283	0.729099636	1.33E-05	0.000793271
Zc3h6	1.817572891	0.862013223	1.93E-05	0.001095524
Zim1	3.499372448	1.807096223	0.000218117	0.008771277
Zkscan14	1.698462628	0.764229475	0.001213465	0.036348363
Zyg11a	1.950435495	0.963796286	0.001615644	0.045586845

Additional file 3-The enriched GO

GOID	Term	Number in list	Number in genome	p-value
Biological_process				
GO:0071346	cellular response to interferon-gamma	6	27	0.00415
GO:0045661	regulation of myoblast differentiation	6	26	0.00343
GO:0050879	multicellular organismal movement	6	22	0.00136
GO:0050881	musculoskeletal movement	6	22	0.00136
GO:0014896	muscle hypertrophy	6	20	0.000835
GO:0003300	cardiac muscle hypertrophy	6	18	0.000457
GO:0014897	striated muscle hypertrophy	6	18	0.000457
GO:0003009	skeletal muscle contraction	6	12	3.82E-05
GO:0006637	acyl-CoA metabolic process	7	58	0.0323
GO:0035383	thioester metabolic process	7	58	0.0323
GO:0006007	glucose catabolic process	7	49	0.0132
GO:0006942	regulation of striated muscle contraction	7	49	0.0132
GO:2001014	regulation of skeletal muscle cell differentiation	7	37	0.00274
GO:0045214	sarcomere organization	7	21	7.87E-05
GO:0046365	monosaccharide catabolic process	8	54	0.00402
GO:0019320	hexose catabolic process	8	52	0.00318
GO:0006641	triglyceride metabolic process	8	48	0.00185
GO:0034341	response to interferon-gamma	8	45	0.00122
GO:0055006	cardiac cell development	8	39	0.000458
GO:0060048	cardiac muscle contraction	8	38	0.000401
GO:0055013	cardiac muscle cell development	8	34	0.000185
GO:0006694	steroid biosynthetic process	9	88	0.0178
GO:0035914	skeletal muscle cell differentiation	9	76	0.00678
GO:0016052	carbohydrate catabolic process	9	73	0.00512
GO:0048747	muscle fiber development	9	71	0.00428
GO:0044724	single-organism carbohydrate catabolic process	9	68	0.00319
GO:0006638	neutral lipid metabolic process	9	60	0.00131
GO:0006639	acylglycerol metabolic process	9	58	0.00104
GO:0003015	heart process	9	47	0.000219
GO:0060047	heart contraction	9	46	0.000188
GO:0016126	sterol biosynthetic process	9	33	1.13E-05
GO:0006695	cholesterol biosynthetic process	9	27	1.98E-06

GO:0051147	regulation of muscle cell differentiation	10	110	0.0194
GO:0046890	regulation of lipid biosynthetic process	10	102	0.0116
GO:0035051	cardiocyte differentiation	10	95	0.00701
GO:0051153	regulation of striated muscle cell differentiation	10	90	0.00469
GO:0055007	cardiac muscle cell differentiation	10	61	0.000232
GO:0055008	cardiac muscle tissue morphogenesis	10	59	0.00018
GO:0048742	regulation of skeletal muscle fiber development	10	52	5.87E-05
GO:1901861	regulation of muscle tissue development	11	132	0.0195
GO:0048634	regulation of muscle organ development	11	131	0.0186
GO:0016202	regulation of striated muscle tissue development	11	130	0.0178
GO:0044262	cellular carbohydrate metabolic process	11	125	0.0132
GO:0048641	regulation of skeletal muscle tissue development	11	80	0.000366
GO:0060415	muscle tissue morphogenesis	11	67	7.45E-05
GO:0051258	protein polymerization	11	45	1.49E-06
GO:0046039	GTP metabolic process	12	167	0.0357
GO:0010876	lipid localization	12	165	0.0323
GO:1901069	guanosine-containing compound catabolic process	12	159	0.0238
GO:0006184	GTP catabolic process	12	155	0.0195
GO:0048644	muscle organ morphogenesis	12	75	3.14E-05
GO:0031032	actomyosin structure organization	12	46	1.47E-07
GO:0030239	myofibril assembly	12	33	2.28E-09
GO:0006006	glucose metabolic process	13	113	0.000319
GO:1901617	organic hydroxy compound biosynthetic process	13	111	0.000264
GO:0006937	regulation of muscle contraction	13	101	0.000104
GO:0016125	sterol metabolic process	13	98	7.49E-05
GO:0008203	cholesterol metabolic process	13	91	3.41E-05
GO:0046165	alcohol biosynthetic process	13	77	5.00E-06
GO:0051186	cofactor metabolic process	14	222	0.0421
GO:0046486	glycerolipid metabolic process	14	201	0.0179
GO:0010927	cellular component assembly involved in morphogenesis	14	150	0.00118
GO:0006631	fatty acid metabolic process	15	249	0.0423
GO:0003007	heart morphogenesis	15	198	0.0051
GO:0008202	steroid metabolic process	15	196	0.0046

GO:0006152	purine nucleoside catabolic process	16	276	0.0417
GO:0046130	purine ribonucleoside catabolic process	16	276	0.0417
GO:0071345	cellular response to cytokine stimulus	16	255	0.0195
GO:0045087	innate immune response	16	241	0.0115
GO:0060538	skeletal muscle organ development	16	172	0.000327
GO:0007519	skeletal muscle tissue development	16	166	0.000219
GO:0005996	monosaccharide metabolic process	16	160	0.000144
GO:0019318	hexose metabolic process	16	143	3.59E-05
GO:0090257	regulation of muscle system process	16	125	5.96E-06
GO:0006941	striated muscle contraction	16	56	4.00E-11
GO:1901658	glycosyl compound catabolic process	17	286	0.0222
GO:0009164	nucleoside catabolic process	17	282	0.0195
GO:0010035	response to inorganic substance	17	281	0.019
GO:0042454	ribonucleoside catabolic process	17	280	0.0186
GO:0003013	circulatory system process	17	247	0.0051
GO:0008015	blood circulation	17	245	0.00469
GO:0043623	cellular protein complex assembly	17	185	0.000197
GO:0048738	cardiac muscle tissue development	17	131	1.98E-06
GO:0055002	striated muscle cell development	17	99	3.36E-08
GO:0034622	cellular macromolecular complex assembly	18	334	0.043
GO:0006066	alcohol metabolic process	18	221	0.000452
GO:0042278	purine nucleoside metabolic process	19	365	0.0448
GO:0046128	purine ribonucleoside metabolic process	19	361	0.0408
GO:1901615	organic hydroxy compound metabolic process	19	304	0.00632
GO:0034762	regulation of transmembrane transport	19	253	0.000714
GO:0034765	regulation of ion transmembrane transport	19	244	0.000452
GO:0055001	muscle cell development	19	127	2.95E-08
GO:1901657	glycosyl compound metabolic process	20	396	0.0463
GO:0009150	purine ribonucleotide metabolic process	20	394	0.0438
GO:0009116	nucleoside metabolic process	20	386	0.0357

GO:0009119	ribonucleoside metabolic process	20	376	0.0267
GO:0044711	single-organism biosynthetic process	21	356	0.00594
GO:0044283	small molecule biosynthetic process	21	344	0.00392
GO:0034097	response to cytokine stimulus	21	332	0.00248
GO:0019693	ribose phosphate metabolic process	22	408	0.0124
GO:0007507	heart development	22	399	0.0097
GO:0044723	single-organism carbohydrate metabolic process	22	341	0.00126
GO:0008610	lipid biosynthetic process	23	370	0.00136
GO:0006955	immune response	25	528	0.0271
GO:0051146	striated muscle cell differentiation	25	211	5.09E-09
GO:0043086	negative regulation of catalytic activity	26	580	0.043
GO:0030036	actin cytoskeleton organization	26	290	6.37E-07
GO:0042692	muscle cell differentiation	26	271	1.70E-07
GO:0005975	carbohydrate metabolic process	27	481	0.0014
GO:0043269	regulation of ion transport	27	434	0.000283
GO:0030029	actin filament-based process	27	311	6.37E-07
GO:0006936	muscle contraction	28	121	1.02E-17
GO:0044057	regulation of system process	29	504	0.000472
GO:0060341	[illegible] localization	30	717	0.0478
GO:0072358	cardiovascular system development	30	695	0.0315
GO:0072359	circulatory system development	30	695	0.0315
GO:0014706	striated muscle tissue development	30	285	8.84E-10
GO:0060537	muscle tissue development	31	301	6.73E-10
GO:0007517	muscle organ development	31	276	6.67E-11
GO:0048646	anatomical structure formation involved in morphogenesis	32	761	0.0315
GO:0044092	negative regulation of molecular function	32	744	0.0217
GO:0006952	defense response	32	716	0.0124
GO:0003012	muscle system process	32	149	2.35E-19
GO:1901700	response to oxygen-containing compound	33	821	0.0498
GO:0070271	protein complex biogenesis	33	639	0.00088
GO:0006461	protein complex assembly	33	637	0.000835
GO:0048878	chemical homeostasis	34	832	0.0333
GO:0044255	cellular lipid metabolic process	35	631	0.000121
GO:0007010	cytoskeleton organization	35	602	4.47E-05

GO:1901135	carbohydrate derivative metabolic process	36	813	0.00622
GO:0065003	macromolecular complex assembly	36	794	0.00415
GO:0019637	organophosphate metabolic process	38	798	0.00104
GO:0061061	muscle structure development	38	396	1.60E-11
GO:0071822	protein complex subunit organization	39	793	0.000422
GO:0006811	ion transport	41	956	0.00406
GO:0043933	macromolecular complex subunit organization	42	986	0.00383
GO:0009888	tissue development	44	1157	0.0222
GO:0022607	cellular component assembly	44	1146	0.019
GO:0006629	lipid metabolic process	45	849	1.13E-05
GO:0002376	immune system process	46	1161	0.00756
GO:0050790	regulation of catalytic activity	50	1414	0.041
GO:0051049	regulation of transport	51	1150	0.000236
GO:0032879	regulation of localization	58	1549	0.00431
GO:0065009	regulation of molecular function	63	1790	0.0102
GO:0010646	regulation of cell communication	67	1979	0.0164
GO:0023051	regulation of signaling	67	1974	0.0154
GO:0044281	process	68	1504	2.15E-06
GO:0048513	organ development	71	2229	0.0478
GO:0065008	regulation of biological quality	71	2087	0.0097
GO:0044765	single-organism transport	72	2062	0.00428
GO:0051239	regulation of multicellular organismal process	72	1989	0.00151
GO:0006950	response to stress	74	2005	0.000663
GO:0006796	phosphate-containing compound metabolic process	76	1799	4.03E-06
GO:0042221	response to chemical stimulus	78	2216	0.00163
GO:0006793	phosphorus metabolic process	80	1847	6.37E-07
GO:0030154	cell differentiation	81	2494	0.0124
GO:0048869	cellular developmental process	83	2609	0.0186
GO:0048523	negative regulation of cellular process	91	2817	0.00594
GO:0044710	single-organism metabolic process	92	2410	1.10E-05
GO:0044767	single-organism developmental process	94	3119	0.0371
GO:0048731	system development	96	3065	0.0102
GO:0048518	positive regulation of biological process	103	3482	0.0357
GO:0048856	anatomical structure development	107	3563	0.0171

GO:0048519	negative regulation of biological process	107	3120	0.000121
GO:0007275	multicellular organismal development	112	3629	0.0046
GO:0016043	cellular component organization	115	3236	7.17E-06
GO:0071840	cellular component organization or biogenesis	116	3373	3.38E-05
GO:0032502	developmental process	123	4020	0.00268
GO:0044237	cellular metabolic process	185	6594	0.00196
GO:0044238	primary metabolic process	194	6803	0.000422
GO:0071704	organic substance metabolic process	198	7030	0.000674
GO:0008152	metabolic process	221	7798	8.05E-05
GO:0050789	regulation of biological process	244	9233	0.00241
GO:0044763	single-organism cellular process	253	9931	0.0167
GO:0065007	biological regulation	256	9631	0.000801
GO:0044699	single-organism process	305	11030	2.88E-07
GO:0009987	cellular process	345	12915	2.46E-07
Cellular_component				
GO:0044291	cell-cell contact zone	6	45	0.048
GO:0014704	intercalated disc	6	41	0.0317
GO:0016460	myosin II complex	6	16	0.000236
GO:0032982	myosin filament	6	14	0.000105
GO:0005861	troponin complex	6	8	1.89E-06
GO:0034705	potassium channel complex	7	54	0.0217
GO:0008076	voltage-gated potassium channel complex	7	53	0.0195
GO:0031430	M band	7	14	3.65E-06
GO:0030315	T-tubule	8	40	0.000548
GO:0065010	extracellular membrane-bounded organelle	9	75	0.00622
GO:0043230	extracellular organelle	9	75	0.00622
GO:0005925	focal adhesion	10	107	0.0163
GO:0031672	A band	10	25	4.48E-08
GO:0030055	cell-substrate junction	12	119	0.00231
GO:0005924	cell-substrate adherens junction	12	112	0.00133
GO:0034703	cation channel complex	12	110	0.00117
GO:0005865	striated muscle thin filament	12	18	2.91E-13
GO:0070161	anchoring junction	13	183	0.0236
GO:0005912	adherens junction	13	169	0.0124
GO:0034702	ion channel complex	13	169	0.0124
GO:0016459	myosin complex	13	62	4.43E-07
GO:0042175	nuclear outer membrane-endoplasmic reticulum membrane network	16	273	0.0378

GO:0042383	sarcolemma	16	105	6.37E-07
GO:0016529	sarcoplasmic reticulum	17	52	6.53E-13
GO:0005874	microtubule	18	331	0.0397
GO:0016528	sarcoplasm	19	60	2.88E-14
GO:0030018	Z disc	25	88	6.52E-18
GO:0031674	I band	32	101	4.83E-25
GO:0015629	actin cytoskeleton	39	348	5.85E-14
GO:0030017	sarcomere	47	138	1.32E-39
GO:0044449	contractile fiber part	48	150	3.76E-39
GO:0030016	myofibril	53	160	6.91E-44
GO:0043292	contractile fiber	54	173	1.69E-43
GO:0005783	endoplasmic reticulum	56	1200	1.67E-05
GO:0044430	cytoskeletal part	59	1200	1.49E-06
GO:0005856	cytoskeleton	84	1755	2.24E-09
GO:0043234	protein complex	101	3306	0.0151
GO:0043232	intracellular non-membrane-bounded organelle	115	2991	1.22E-07
GO:0043228	non-membrane-bounded organelle	115	2991	1.22E-07
GO:0044446	intracellular organelle part	134	4431	0.00181
GO:0044422	organelle part	161	4550	5.57E-09
GO:0044444	cytoplasmic part	214	5646	6.84E-17
GO:0043231	intracellular membrane-bounded organelle	240	9048	0.00235
GO:0043227	membrane-bounded organelle	246	9093	0.000412
GO:0043229	intracellular organelle	303	10046	3.19E-12
GO:0005737	cytoplasm	303	8676	1.53E-22
GO:0043226	organelle	306	10089	7.85E-13
GO:0044424	intracellular part	347	11468	2.36E-16
GO:0005622	intracellular	349	11616	5.28E-16
GO:0005623	cell	391	14395	1.32E-11
GO:0044464	cell part	391	14395	1.32E-11
Molecular_function				
GO:0051393	alpha-actinin binding	6	21	0.00111
GO:0016877	ligase activity, forming carbon-sulfur bonds	7	27	0.000414
GO:0042805	actinin binding	7	25	0.00025
GO:0008307	structural constituent of muscle	7	19	3.85E-05
GO:0031432	titin binding	7	8	1.71E-08
GO:0005516	calmodulin binding	12	148	0.0139
GO:0005200	structural constituent of cytoskeleton	13	44	5.35E-09
GO:0022843	voltage-gated cation channel activity	14	124	0.000176
GO:0003924	GTPase activity	15	186	0.00274

GO:0022832	voltage-gated channel activity	15	171	0.00117
GO:0005244	voltage-gated ion channel activity	15	171	0.00117
GO:0019001	guanyl nucleotide binding	20	364	0.0186
GO:0032561	guanyl ribonucleotide binding	20	364	0.0186
GO:0005525	GTP binding	20	346	0.0109
GO:0005198	structural molecule activity	25	421	0.00125
GO:0042803	protein homodimerization activity	27	616	0.0462
GO:0003779	actin binding	37	340	7.85E-13
GO:0046983	protein dimerization activity	38	993	0.0481
GO:0042802	identical protein binding	43	935	0.000629
GO:0005524	ATP binding	54	1433	0.00659
GO:0032559	adenyl ribonucleotide binding	56	1464	0.00353
GO:0030554	adenyl nucleotide binding	57	1480	0.00255
GO:0008092	cytoskeletal protein binding	60	657	6.52E-18
GO:0035639	purine ribonucleoside triphosphate binding	74	1749	5.89E-06
GO:0032553	ribonucleotide binding	76	1804	4.47E-06
GO:0032555	purine ribonucleotide binding	76	1789	3.28E-06
GO:0001882	nucleoside binding	76	1775	2.41E-06
GO:0001883	purine nucleoside binding	76	1765	1.98E-06
GO:0032550	purine ribonucleoside binding	76	1762	1.98E-06
GO:0032549	ribonucleoside binding	76	1765	1.98E-06
GO:0017076	purine nucleotide binding	77	1806	2.29E-06
GO:0036094	small molecule binding	84	2351	0.000466
GO:1901265	nucleoside phosphate binding	84	2207	4.92E-05
GO:0000166	nucleotide binding	84	2207	4.92E-05
GO:0043168	anion binding	91	2309	3.28E-06
GO:0003824	catalytic activity	157	5299	0.000801
GO:0043167	ion binding	166	5366	3.31E-05
GO:0005515	protein binding	224	6507	4.89E-13
GO:0005488	binding	334	11090	1.05E-14

Additional file 4-The enriched signaling pathways

Term	Id	P-Value	Corrected P-Value	Genes
Hypertrophic cardiomyopathy (HCM)	mmu05410	2.72E-07	4.03E-05	Cacng1|Myh7|Cacna1s|Ttn|Sgcg|Tnnc1|Myl2|Agt|Sgca|Itgb6|Cacng6|
Dilated cardiomyopathy	mmu05414	4.80E-06	0.00035554	Cacng1|Myh7|Cacna1s|Ttn|Sgcg|Tnnc1|Myl2|Sgca|Itgb6|Cacng6|
Glycolysis / Gluconeogenesis	mmu00010	2.98E-05	0.001470744	Acss2|Pfkm|Pgam2|Fbp2|Hkdc1|Eno3|Hk2|Pck1|
Arrhythmogenic right ventricular cardiomyopathy (ARVC)	mmu05412	4.48E-05	0.001657221	Cacng1|Cacna1s|Actn3|Sgcg|Actn2|Sgca|Itgb6|Cacng6|
Cardiac muscle contraction	mmu04260	0.000583609	0.017274833	Cox6a2|Myh7|Cacna1s|Cacng1|Tnnc1|Myl2|Cacng6|
Tight junction	mmu04530	0.000775363	0.019125629	Myh7|Prkcq|Myh4|Actn3|Actn2|Myh2|Myh1|Myl2|Mylpf|
Carbon metabolism - Mus musculus (mouse)	mmu01200	0.001775289	0.036211823	Acss2|Pfkm|Pgam2|Fbp2|Hkdc1|Eno3|Hk2|
Calcium signaling pathway	mmu04020	0.001957396	0.036211823	Mylk4|Slc8a3|Ryr1|Tnnc1|Cacna1s|Camk2b|Camk2a|Tnnc2|Mylk2|Atp2a1
Butirosin and neomycin biosynthesis	mmu00524	0.002942709	0.046599792	Hkdc1|Hk2|
PPAR signaling pathway	mmu03320	0.003148635	0.046599792	Acsl6|Acsl3|Scd2|Angptl4|Fabp3|Pck1|
HIF-1 signaling pathway - Mus musculus (mouse)	mmu04066	0.003564412	0.047957539	Hkdc1|Camk2b|Camk2a|Egf|Eno3|Hk2|Serpine1|

Additional file 5-DEGs related to lipid metabolis

Id	FoldChange(HSvsNS)	Log2FoldChange	P-val	P-adj
Acaa1b	1.703642063	0.768622256	1.83E-07	1.68E-05
Acot3	3.245579893	1.69847627	0.000320011	0.012045231
Acsl3	0.470225673	-1.088574787	4.41E-10	6.44E-08
Acsl6	4.403660657	2.1387033	0.001764891	0.048845967
Acsm3	2.160677946	1.111484051	9.64E-09	1.13E-06
Acsm5	1.9362304	0.953250635	4.62E-08	4.85E-06
Acss2	0.427374869	-1.226426019	1.89E-05	0.00108027
Acss3	1.537650869	0.620727969	0.000201586	0.0082475
Adap1	0.314397841	-1.669336783	2.83E-19	1.30E-16
Agpat2	0.582819807	-0.778878188	1.40E-05	0.000817994
Agt	2.41311992	1.270899612	1.60E-17	5.69E-15
Angptl4	2.504627475	1.324596041	0.000499978	0.017360858
Ank1	2.452852657	1.294460574	0.001453901	0.042356696
Ankrd23	4.534677567	2.180999973	1.65E-12	3.30E-10
Atp10a	0.577323798	-0.792547398	6.04E-06	0.000390454
Atp2a1	61.61272023	5.945156328	2.93E-18	1.17E-15
Barx2	10.87150535	3.442479815	0.000162287	0.006878906
Cacna1s	31.88788129	4.99493634	1.56E-20	8.91E-18
Camk2a	4.850225556	2.27805184	3.56E-05	0.001875075
Camk2b	4.439348046	2.150347821	1.25E-11	2.30E-09
Cav3	9.23868415	3.207687386	9.20E-09	1.08E-06
Ccl12	0.32165696	-1.63640519	9.26E-05	0.004292562
Cd14	1.841489535	0.880873198	0.001469077	0.042653177
Ces1d	1.568087016	0.64900562	0.000189406	0.007868902
Chsy1	0.351174248	-1.509741039	2.97E-10	4.54E-08
Cpt1b	1.851251718	0.888501075	0.000295461	0.011301971
Csrp3	34.64490997	5.114571502	3.52E-20	1.95E-17
Cyp2e1	3.245771191	1.698561301	1.17E-17	4.22E-15
Dfna5	0.453149977	-1.141939483	6.44E-08	6.48E-06
Dgat2	0.488206844	-1.034435576	6.29E-07	5.10E-05
Dhcr24	0.667410904	-0.583352838	9.03E-05	0.004208378
Dhcr7	0.510154947	-0.970992597	0.001535019	0.043885418
Egf	3.06089373	1.613952957	1.99E-06	0.000142172
Eno3	6.321589633	2.660287386	8.08E-55	2.17E-51
Ephx2	1.528657333	0.612265045	2.61E-05	0.001417061
Fabp3	6.489993857	2.698217113	5.26E-13	1.09E-10
Fabp5	0.542316324	-0.882793499	2.25E-08	2.46E-06
Fbp2	6.397738568	2.677562041	5.34E-09	6.48E-07
Fitm1	43.51412923	5.443412021	1.07E-15	3.09E-13
G6pdx	0.603804673	-0.727846172	6.83E-06	0.000436023
Grb7	0.341620812	-1.549532226	6.10E-08	6.17E-06
Gulp1	1.652140921	0.724336749	0.000269903	0.010561045
Hhatl	7.263239489	2.86061315	1.10E-08	1.27E-06
Hk2	0.477435966	-1.066620844	1.92E-05	0.001094309
Hkdc1	0.048988045	-4.351426469	4.38E-05	0.002212378
Hmgcr	0.490607258	-1.027359518	3.13E-09	3.98E-07
Hp	1.562198444	0.643577728	0.000305833	0.011674992
Insig1	0.487810662	-1.035606804	5.60E-10	8.05E-08
Klf11	1.908750634	0.932628636	4.54E-05	0.002277981

Ky	4.666018078	2.222191897	1.34E-13	2.87E-11
Ldlr	0.398120594	-1.328722594	1.75E-19	8.22E-17
Lep	0.411466132	-1.281154407	1.55E-16	5.03E-14
Lpgat1	0.613851664	-0.704038022	4.24E-05	0.002158143
Mb	6.652456123	2.73388709	9.26E-46	1.94E-42
Mef2c	1.598848519	0.677033259	3.40E-05	0.001801594
Mid1ip1	0.354740915	-1.495162358	2.16E-11	3.79E-09
Mogat2	0.408532907	-1.291475804	1.55E-12	3.14E-10
Murc	11.28513304	3.496351521	2.90E-08	3.14E-06
Mvd	0.354129602	-1.49765065	1.09E-07	1.06E-05
Mvk	0.578143584	-0.79050026	1.04E-05	0.000633019
Neb	26.57163363	4.731815023	5.12E-20	2.63E-17
Neu2	13.59760673	3.765280845	0.000443519	0.015719432
Neurl1a	2.543523341	1.346828334	1.53E-06	0.000112694
Nrip1	1.578803694	0.6588318	6.53E-05	0.003168659
Obscn	39.67271926	5.310075382	3.90E-123	3.67E-119
Osm	0.17556744	-2.509902785	0.001169594	0.035331868
Pck1	5.352804386	2.420294932	4.68E-13	9.79E-11
Pdk4	2.481568434	1.31125224	1.82E-15	5.04E-13
Pla2g4e	23.13139198	4.531780181	8.17E-06	0.000511078
Plin2	0.474884254	-1.074352175	2.64E-05	0.001427753
Pmvk	0.518691741	-0.947050696	2.72E-07	2.36E-05
Pnpla3	0.561840469	-0.831767551	1.71E-06	0.000124409
Ppp1r14c	35.8094063	5.162266695	1.50E-12	3.07E-10
Ppp1r3a	33.84142622	5.080718466	2.50E-07	2.20E-05
Ppp1r3b	0.282726959	-1.822518638	1.50E-10	2.32E-08
Ppp2r5b	0.387383914	-1.368164049	3.31E-17	1.15E-14
Pygm	7.351196527	2.877979091	1.12E-21	7.52E-19
Raf1	0.608985941	-0.715519173	8.81E-07	6.82E-05
Rbm24	2.709218186	1.437876585	1.59E-06	0.00011708
Rhob	0.551368531	-0.858911167	5.26E-08	5.47E-06
Scd2	0.423367545	-1.240017416	0.000271602	0.010561045
Serinc2	6.259543017	2.646057336	0.000125565	0.005508459
Serpine1	0.402500131	-1.312938842	1.29E-07	1.24E-05
St3gal6	2.199473174	1.137158006	1.30E-06	9.74E-05
Tcap	36.67709286	5.196807386	5.96E-91	2.80E-87
Thrsp	0.563493183	-0.827529937	0.001381785	0.040569718
Tnfrsf12a	0.448745967	-1.156029121	0.001470877	0.042653177
Ucp3	0.538996277	-0.891652786	2.68E-06	0.000187926
Utp14b	0.444069823	-1.17114156	5.47E-05	0.002686704
Vnn1	2.66322743	1.413175635	0.000159319	0.006799058
Xirp1	2.347698017	1.231246847	2.04E-05	0.001155181

Additional file 6-DEGs related to ion

Ca2+	
Gene	FoldChange(HSvsNS)
Acta1	35.77756326
Actn2	28.16717385
Actn3	50.74742164
Agt	2.41311992
Akap5	0.57171703
Atp2a1	61.61272023
Cacna1s	31.88788129
Cacng1	14.40988167
Camk2a	4.850225556
Camk2b	4.439348046
Capn11	17.21370618
Capn3	4.478474739
Casq1	19.63077632
Cav3	9.23868415
Cmklr1	0.48129197
Csrp3	34.64490997
Egf	3.06089373
Hrc	13.24996283
Itgb1bp2	6.135697666
Mef2c	1.598848519
Msh5	3.296746846
Myh1	33.8342002
Myh2	26.95474783
Myh4	110.5217934
Myh7	15.9706915
Myl1	2.732810156
Myl2	10.2729991
Mylk2	12.85079122
Mylk4	19.5421513
Mylpf	36.92063239
Ncan	0.401207839
Prkcq	3.399059251
Pvalb	112.1819827
Pygm	7.351196527
Ryr1	35.24234969
Sgca	2.998961714
Slc8a3	3.820715446
Sln	29.86835105
Sypl2	10.10970154

Tnnc1	2.616477751
Tnnc2	44.98500273
Tnni1	4.789102742
Tnni2	64.85067014
Tnnt1	4.372952242
Tnnt3	58.90112487
Trdn	7.673392274
Ttn	56.77753369
Na+	
Gene	FoldChange(HSvsNS)
Scn4a	19.88152714
Scn4b	3.657473142
Slc1a3	2.217125658
Slc8a3	3.820715446
Scn1b	1.658694564
Cav3	9.23868415
K+	
Gene	FoldChange(HSvsNS)
Kcna7	11.08628997
Kcnc1	5.069325209
Kcnc4	2.902571169
Kcng4	0.353080818
Kcnj11	8.665119074
Kcnj12	2.818000212
Tmem38a	3.015912239
Cntnap2	23.624

Additional file 7-Genes related to RAS,common genes between RAS and lipid metabolism

Gene related to RAS	FoldChange(HSvsNS)
Acta1	35.77756326
Agt	2.41311992
Angpt1	1.699811693
Angptl4	2.504627475
Actn2	28.16717385
Actn3	50.74742164
Camk2a	4.850225556
Camk2b	4.439348046
Egf	3.06089373
Eno3	6.321589633
Pla2g4e	23.13139198
Prkcq	3.399059251
Ccl12	0.32165696
Grb7	0.341620812
Hk2	0.477435966
Hkdc1	0.048988045
Raf1	0.608985941
Serpine1	0.402500131
Tnfrsf12a	0.448745967
Common genes between RAS and lipid metabolism	FoldChange(HSvsNS)
Angptl4	2.504627475
Pla2g4e	23.13139198
Ccl12	0.32165696
Grb7	0.341620812
Hk2	0.477435966
Hkdc1	0.048988045
Raf1	0.608985941
Serpine1	0.402500131
Tnfrsf12a	0.448745967
Agt	2.41311992
Egf	3.06089373
Camk2a	4.850225556
Camk2b	4.439348046
